# Fuzzy-based propagation of prior knowledge to improve large-scale image analysis pipelines

**DOI:** 10.1371/journal.pone.0187535

**Published:** 2017-11-02

**Authors:** Johannes Stegmaier, Ralf Mikut

**Affiliations:** Institute for Applied Computer Science, Karlsruhe Institute of Technology, Eggenstein-Leopoldshafen, Germany; Pennsylvania State Hershey College of Medicine, UNITED STATES

## Abstract

Many automatically analyzable scientific questions are well-posed and a variety of information about expected outcomes is available *a priori*. Although often neglected, this prior knowledge can be systematically exploited to make automated analysis operations sensitive to a desired phenomenon or to evaluate extracted content with respect to this prior knowledge. For instance, the performance of processing operators can be greatly enhanced by a more focused detection strategy and by direct information about the ambiguity inherent in the extracted data. We present a new concept that increases the result quality awareness of image analysis operators by estimating and distributing the degree of uncertainty involved in their output based on prior knowledge. This allows the use of simple processing operators that are suitable for analyzing large-scale spatiotemporal (3D+t) microscopy images without compromising result quality. On the foundation of fuzzy set theory, we transform available prior knowledge into a mathematical representation and extensively use it to enhance the result quality of various processing operators. These concepts are illustrated on a typical bioimage analysis pipeline comprised of seed point detection, segmentation, multiview fusion and tracking. The functionality of the proposed approach is further validated on a comprehensive simulated 3D+t benchmark data set that mimics embryonic development and on large-scale light-sheet microscopy data of a zebrafish embryo. The general concept introduced in this contribution represents a new approach to efficiently exploit prior knowledge to improve the result quality of image analysis pipelines. The generality of the concept makes it applicable to practically any field with processing strategies that are arranged as linear pipelines. The automated analysis of terabyte-scale microscopy data will especially benefit from sophisticated and efficient algorithms that enable a quantitative and fast readout.

## Introduction

Available prior knowledge is often not sufficiently considered by automatic processing pipelines. Consequently, a great amount of potentially useful extra information remains unused. Particularly in the domains of image processing and image analysis, the visual analysis of acquired image data offers a large repository of usable *a priori* information that can often easily be verbalized by experts of the respective application fields. In contrast to this, examples of the successful incorporation of prior knowledge are, *e*.*g*., the approaches described in [1, 2]; these make use of information about the expected object number as well as their associated physical size in order to adjust and improve seed point detection algorithms. Analogously, properties such as size, shape, geometry, intensity distributions and the like can be used to improve the performance of image segmentation algorithms [3–5]. Such prior knowledge is often embedded into the algorithms via shape penalization terms that are appended to the energy functional of a graph-cut [4, 6] or a level-set segmentation [7] or by generalized Hough transforms that can detect arbitrary shapes [8]. Object properties such as size, shape and movement dynamics can also be used to formulate efficient correction heuristics for object tracking algorithms [9–11].

A great, but often underrated potential for algorithmic improvements lies in the estimation of uncertainties of the automatically produced results and should ideally be considered by subsequent processing steps [12]. On the pixel level, this uncertainty can be used to assess the information quality of a single pixel due to sensor imperfections or temperature dependence [12, 13]. Furthermore, the localization uncertainty of geometric features such as corners, centroids, edges and lines in images was assessed in [14–17]. An approach to evaluate the quality of image registration algorithms was presented in [18]. Apart from quality assessment, uncertainty quantification also plays a role in areas such as face recognition and other biometric technologies [19–21], the tracking of shapes in ultrasound images [22] or to evaluate the impact of noisy measurements on the validity of diagnosis results [23]. An uncertainty formulation based on fuzzy set theory was employed to perform pixel- or object-based classification tasks [24–26]. A further possibility of exploiting uncertainty information is to optimize parameter values of a respective operator in a feedback fashion such that the outcome minimizes a previously defined optimization criterion as demonstrated in [27, 28]. Another example is the improvement of a graph-based watershed implementation, where uncertainties are used to assess the influence of individual edges on the final segmentation outcome [29].

Hitherto, however, a uniform approach to systematically transform, embed and use the available prior knowledge to improve both existing and new algorithms has been missing. Although the sequential arrangement of processing operators is a widely used concept in image analysis, results propagated through the pipelines are mostly not assessed by the individual pipeline components with respect to their result quality. Thus, errors made in early processing steps tend to accumulate and may negatively affect the final result quality. Additionally, many existing methods for processing tasks such as seed point detection, segmentation and tracking are often not directly applicable to large-scale 3D+t data sets due to enormous memory or computation time demands.

Throughout the present contribution, uncertainty is considered to be imperfect knowledge about the validity of a piece of extracted information produced by an image analysis operator with respect to available prior knowledge. We use the term uncertainty propagation to refer to sharing information about result quality among different pipeline components and use this information to derive efficient improvement heuristics to enhance the final outcome of the pipelines [30, 31].

The work presented in this paper contains parts of a recently published PhD thesis by one of the authors (J. Stegmaier) [32] and is based on our previous concept paper [31], where we sketched the concept of using fuzzy set theory to transform available prior knowledge into a mathematical representation. A list of symbols is provided in S1 Table. Here, we briefly recapitulate the theoretical framework and perform an extensive validation of the proposed concepts to enhance the performance and to improve the results of image analysis operators by data filtering, uncertainty propagation and explicit exploitation of information uncertainty. In particular, we extend an exemplary image analysis pipeline comprised of seed point detection, segmentation, multiview fusion and tracking with uncertainty handling. We demonstrate how simple processing operators can be extended with uncertainty handling to improve large-scale analyses of 3D+t microscopy images. All methods are quantitatively validated on a comprehensive simulated validation benchmark data set that mimics embryonic development and is inspired by epiboly movements of zebrafish embryos. Moreover, we show qualitative results obtained with the presented framework on large-scale light-sheet microscopy data of developing zebrafish embryos.

## Methods

### Uncertainty propagation in image analysis pipelines

#### The image analysis pipeline concept

**Fig 1:**
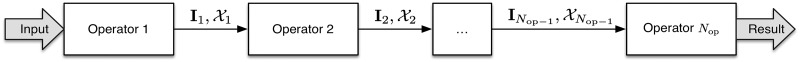
General image analysis pipeline comprised of *N*_op_ sequentially arranged processing operators. Each operator directly depends on the quality of the input images (I_*_) or features ($X_{*}$) provided by its predecessor (adapted from [31]).

Most image analysis pipelines make use of multiple processing operators that are arranged as a linear processing pipeline and perform specialized tasks, such as improving or transforming the image signal, or extracting information from the images (Fig 1). The *N*_op_ sequentially connected operators receive either an input image (denoted by **I**_*i*_), extracted features (denoted by $X_{i}$) or both from their preceding processing operator with *i* ∈ {1, …, *N*_op_} being the ID of the operator.

The output set $X_{i}$ of processing operator *i* is an (*N*_*i*_ × *N*_f,*i*_) matrix with *N*_*i*_ data tuples and *N*_f,*i*_ features. For processing operators without any feature output, $X_{i}$ is an empty matrix and only the processed image is passed to the next operator.

#### Identification of suitable prior knowledge

Prior knowledge can be obtained from expert knowledge, literature, experimental evidence or knowledge databases. Through visual analysis of acquired data, experts can often easily identify recurring patterns, intensity properties or the appearance of objects and can describe these in natural language. An exemplary overview of such prior information derived from microscopy images is summarized in Table 1. Here, prior information is listed in bottom-up order, *i*.*e*. starting at the acquisition stage via the content of a single image, through to the comparison among time series of images or features. Naturally, the listing is not exhaustive and suitable features have to be carefully selected to match the underlying image material and analysis problem. In the following sections, the presented natural language expressions will be used to transform the prior knowledge of different sources to a consistent mathematical representation using the concept of fuzzy sets.

**Table 1:** Prior knowledge for 3D+t image analysis and exemplary natural language expressions.

Source	Description	Example
Image Acquisition	Acquisition-specific prior knowledge such as illumination conditions, detection path, image resolution, physical spacing of voxels, high-quality image regions, point spread function (PSF) or the detection path.	Image quality decreases from … to …
Intensity	Signal-dependent information like the intensity range, time-variant characteristics of objects (*e*.*g*., photobleaching in fluorescence microscopy), signal-to-noise ratio and global statistical properties of the image intensity values.	Valid objects are brighter than …
Localization	Positional information of the objects or object properties in absolute image coordinates. Furthermore, localization of extracted properties or objects relative to each other can be used to define neighborhood relations.	Object type … only appears close to location …
Spatial Extent	Object properties such as size, volume, principal components, convex hull extents or bounding volumes.	Object type … is larger than … but smaller than …
Geometry	Geometrical properties like dimensionality, symmetry, shape, proportions and relative localization of features within an object.	Object type … has a line-like shape with a central symmetry axis.
Morphology	Combination of intensity-based and geometrical properties, *e*.*g*., to link information about patterning, texture, structure and color to geometrical properties such as shape and symmetry.	Object … is spherical, bright and has a textured surface.
Object Interaction	Characterization of between-object properties like clustering, adhesion, repulsion, division or regional density changes.	Object behavior … rather appears in dense regions.
Spatio-Temporal Coherence	Dynamically changing quantities such as object growth, movement direction, speed, object appearance and disappearance.	Object moves maximally … pixels between two subsequent frames.

#### Quantifying prior knowledge using fuzzy set membership functions

To transform the prior knowledge presented in Table 1 into a mathematical representation, we make use of fuzzy sets that have been introduced by Zadeh in 1965 [33]. The simple yet powerful concept of fuzzy sets enables natural language descriptions of observed phenomena to be easily mapped to a numerical representation and can also be used to model vague knowledge or partially true statements. While estimating density functions for a probabilistic framework might be cumbersome, time consuming or simply not possible due to a lack of data, fuzzy sets can be easily parameterized based on prior knowledge that is usually available from (biological) experts in the field or can be derived from a few representative data points. Additionally, valid objects may exhibit a different size, shape or appearance but are still equally correct. Thus, plateau regions in trapezoidal fuzzy set membership functions offer a convenient and practically motivated way of modeling such diversity among valid objects. Last but not least, the close connection to natural language expressions makes fuzzy set theory easily understandable, even without a strong mathematical background.

**Fig 2:**
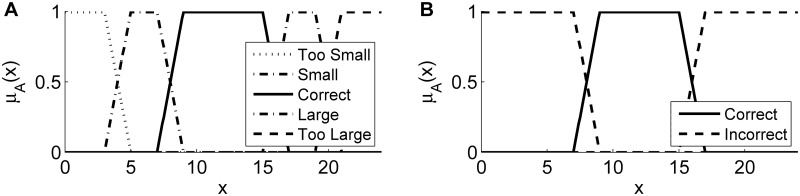
Different possibilities to partition the input space of a feature *x* using trapezoidal membership functions. In (A), each of the linguistic terms has a separate fuzzy set and (B) shows a reduced version with only two fuzzy sets that correspond to the desired class and its complement. In (B), different possibilities to summarize the correct objects arise. Besides restricting the class to the correct set as done in (B), the correct fuzzy set could be extended by the potentially useful classes (*Small* and *Large*). However, the appropriate formulation has to be chosen application dependent.

Analogous to the characteristic function of a classical set, a fuzzy set A can be defined by its associated membership function (MBF) $\mu_{A}:X\mapsto \left[0,1\right]$ that maps each element of a universe of discourse X to a value in the range [0, 1] [33]. This assigned value in turn directly reflects the fuzzy set membership degree (FSMD) of the respective element to the fuzzy set A. The special cases $\mu_{A}\left(x\right)=1$ and $\mu_{A}\left(x\right)=0$ indicate that *x* is fully included or not part of the fuzzy set A, respectively [34]. The most common membership functions used in practice are trapezoidal membership functions, which can be parameterized to model singletons, triangular and rectangular MBFs. A trapezoidal membership function can be formulated as

$$\begin{array}{c} \mu_{A}\left(x,\theta \right)=\max(\min(\frac{x-a}{b-a},1,\frac{d-x}{d-c}),0), \end{array}$$ (1)

with the parameter vector ***θ*** = (*a*, *b*, *c*, *d*)^⊤^ that is used to control the start and end points of the respective transition regions. Here, we make use of a standard partition, *i*.*e*., maximally two neighboring fuzzy sets overlap and have non-zero membership values for a certain value of *x* and the respective membership degrees for any input value of *x* sum up to 1 (Fig 2).

As an example, consider an object detection algorithm where a size-based feature of each object serves as an indicator of its appropriateness. As described in [35], the linguistic terms could be determined by five possible outcomes, where the extracted size feature …

1. … perfectly matches the expected value (*Correct*).
2. … is smaller than expected but might be useful (*Small*).
3. … is larger than expected but might contain useful information (*Large*).
4. … is too small and not useful (*Too Small*, *e*.*g*., noise or artifacts).
5. … is too large and not useful (*Too Large*, *e*.*g*., segments in background regions).

Available prior knowledge can be used to determine the parameterization of the associated fuzzy sets and an exemplary standard partition is shown in Fig 2A. If only one outcome of the operators is of importance (*e*.*g*., Case 1 in the above-mentioned example), it is possible to use only one linguistic term and to aggregate all other cases by its complement (Fig 2B). We use $\mu_{A_{ifl}}:R\to \left[0,1\right]$ to denote the fuzzy set membership function for image analysis operator *i*, feature *f* ∈ {1, …, *N*_f,*i*_} and linguistic term *l* ∈ {1, …, *N*_l_}. Thus, the *n*-th data tuple produced by operator *i* obtains the FSMD value $\mu_{A_{ifl}}\left(x_{i}\left[n,f\right]\right)$ to the fuzzy set $A_{ifl}$ for each feature *f* and each linguistic term *l*.

#### Combination of fuzzy set membership functions

Fuzzy set membership degree values of multiple features that characterize a linguistic term (*e*.*g*., if an object of interest is bright and elongated at the same time) can be combined using a fuzzy pendant to a logical conjunction [30]. A conjunction of *N*_f,*i*_ fuzzy set membership functions for linguistic term *l* can be defined using the general t-norm operator ∩ (triangular norm):

$$\begin{array}{c} \mu_{\text{lc},A_{il}}\left(x_{i}\left[n\right]\right)=\underset{f=1,\ldots N_{\text{f},i}}{\cap }\left(\mu_{A_{ifl}}\left(x_{i}\left[n,f\right]\right)\right). \end{array}$$ (2)

Features that should not contribute to the combined fuzzy set membership function can be disabled by setting the corresponding MBFs to the constant value 1 (identity element of the conjunction) and the complement of the combined linguistic term is simply given by $1-\mu_{\text{lc},A_{il}}\left(x_{i}\left[n\right]\right)$ as illustrated in Fig 2B. Common fuzzy t-norm operators are the multiplication, the bounded difference and the minimum operator [36]. In S1 Note, we provide an overview of the three major t-norm/t-conorm pairs and discuss the respective advantages and drawbacks of the different operators.

#### Uncertainty propagation in image analysis pipelines

We use the FSMD values associated with each data tuple to perform a feed-forward propagation of the reliability of extracted data to downstream operators. For each data vector that is produced by operator *i*, we calculate the degree of membership to the respective fuzzy sets and append it to the feature output $X_{i}$. If only a classification into correct vs. incorrect objects needs to be performed (Fig 2B) or if a linguistic term is described by a combination of different fuzzy sets (Eq (2)), a single FSMD value is appended per data tuple. Besides using the FSMD values for object classification, the gradual membership values to one or the other class can explicitly be used to perform object filtering, weighted object fusion, extended information propagation or to resolve ambiguities.

#### Uncertainty-based object rejection

The first application of the uncertainty framework is to filter the extracted output information $X_{i}$ produced by an operator *i* using thresholds *α*_*il*_ ∈ [0, 1]. According to the FSMD values $\mu_{\text{lc},A_{il}}\left(x_{i}\left[n\right]\right)$ calculated for each data tuple $x_{i}\left[n\right]\in X_{i}$, **x**_*i*_[*n*] is only passed to the next pipeline component if $\mu_{\text{lc},A_{il}}\left(x_{i}\left[n\right]\right)\geq \alpha_{il}$ for the membership to a desired set. The reduced set which serves as input for operator *i* + 1 is denoted by ${\tilde{X}}_{i}$. To keep all extracted information, the threshold is set to *α*_*il*_ = 0. In contrast, when *α*_*il*_ = 1, no uncertain information is passed to operator *i* + 1. Based on application specific criteria or object properties, this FSMD-based object rejection readily allows false positive detections to be filtered out, as demonstrated in the following sections on seed point detection and segmentation, respectively.

#### Extended information propagation to compensate operator flaws

Second, we allow operators to fall back on information of penultimate processing steps if predecessors do not deliver good results. For instance, if an operator *i* fails to sufficiently extract information from its provided input data (*e*.*g*., missing, merged or misshapen objects), it can inform downstream operators about these flawed results. Using a second threshold *β*_*il*_ ∈ [*α*_*il*_, 1] for each operator, the FSMD level below which the information of the previous steps should be additionally propagated can be controlled. This means that instead of only forwarding the *α*_*il*_-filtered set ${\tilde{X}}_{i}\subseteq X_{i}$ produced by operator *i* to operator *i* + 1 a set $\Omega_{i}={\tilde{X}}_{i}\cup {\tilde{\Omega }}_{i-1}$ with *i* ≥ 2 and $\Omega_{1}={\tilde{X}}_{1}$ is passed through the pipeline.

${\tilde{\Omega }}_{i-1}$ represents the subset of elements in Ω_*i*−1_ which were not successfully transferred into useful information by operator *i*, *i*.*e*., elements **x**_*i*−1_[*n*] ∈ Ω_*i*−1_ that generated output $x_{i}\left[n\right]\in {\tilde{X}}_{i}$ with $\alpha_{il}\leq \mu_{\text{lc},A_{il}}\left(x_{i}\left[n\right]\right)<\beta_{il}$. Such elements characterize information of operator *i* − 1 that might be useful in later steps to correct flawed results of operator *i*. If *β*_*il*_ = 1 all information of *i* − 1 that produced an uncertain outcome is propagated to the successor *i* + 1. If *β*_*il*_ = *α*_*il*_ only the information $\tilde{X_{i}}$ produced by operator *i* is propagated. In the current version of the framework, the respective processing operators are responsible for calculating Ω_*i*_ and the respective FSMD values.

This approach successfully resolved tracking conflicts that originated from under-segmentation errors as described in the results section.

#### Resolve ambiguities using propagated uncertainty

In addition to filtering and propagating the operator information within the pipeline, uncertainty information can explicitly be used by the processing operators to improve their results. Depending on the degree of uncertainty of information, parameters or even whole processing methods can be adapted if needed. Although the adaptations required by a particular algorithm cannot be generalized, we present two potential applications: the fusion of redundant seed points and the correction of under-segmentation errors.

**Fig 3:**
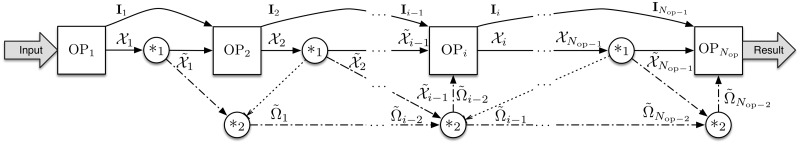
Extended image analysis pipeline concept. Extracted output information of each operator can be filtered according to its uncertainty (*_1_), operators can access information produced by penultimate predecessors (*_2_) and processing operators can specifically adjust their processing behavior based on FSMD values of extracted information. Solid lines indicate the main information flow, dash-dotted lines the propagation of previously calculated results and dotted lines emphasize influence on the selection of propagated information. In addition to the flow of extracted features ($X_{*}$, Ω_*_, ${\tilde{X}}_{*}$, ${\tilde{\Omega }}_{*}$), the operators may pass processed images, image parts or forward the input image (I_*_) to the subsequent processing operator.

The general scheme for the proposed uncertainty propagation framework is summarized in Fig 3 and is applied to an exemplary image analysis pipeline in the next sections.

### Extending and enhancing algorithms with uncertainty treatment

Based on the general concept presented in the previous section, we applied it to an exemplary image analysis pipeline comprised of seed point detection, segmentation, multiview fusion and object tracking. For each operator, FSMD values were estimated based on prior knowledge and used for algorithmic improvements where possible. We used a simulated benchmark data set that mimicked 3D microscopy images containing fluorescently labeled nuclei of an artificial embryo (S1 and S2 Videos). The benchmark was inspired by the epiboly movements happening in early zebrafish development. After the blastula stage is complete, cells located at the animal pole of the embryo spread and thin toward the vegetal pole, yielding cell layers that entirely cover the sphere-shaped yolk cell [37].

The use of simulated image data for validation is advantageous because it allows a single comprehensive data set for the validation of all pipeline components. This enables specific bottlenecks or error sources in the processing pipelines to be uncovered, instead of testing each of the pipeline components separately on different benchmarks. Furthermore, different acquisition deficiencies such as different point-spread-functions, decreasing signal-to-noise ratios or multiview acquisition deficiencies can be simulated. The immediate availability of a reliable ground truth enables a quantitative validation without the bias observed for manually annotated benchmark data that suffers from intra- and inter-expert variability. As the simulated benchmark is close to the target application of the pipeline, namely quantitatively analyzing terabyte-scale 3D+t fluorescence microscopy images, the developed concepts and algorithms can easily be put into practice, *e*.*g*., for false positive reduction of a segmentation algorithm or for segmentation-based multiview fusion [32, 37].

Details on benchmark generation can be found in S2 Note, S1 Fig and [38]. Abbreviations for the different algorithms are given in round brackets and a quantitative comparison of the result quality is provided in the results section.

#### Seed point detection

In [39], a blob detection method based on the Laplacian-of-Gaussian (LoG) maximum intensity projection was used to localize fluorescently labeled cellular nuclei in 3D microscopy images. A 3D input image was filtered with differently scaled LoG filters with standard deviations *σ* matching the expected object radius *r* using the relation $\sigma =r/\sqrt{2}$. Subsequently, the 3D maximum projection of these LoG-filtered images was formed and local extrema were extracted from this projection image (LoGSM). Although the proposed method worked well in many scenarios, it frequently missed objects that did not exhibit a strict local maximum due to an intensity plateau (*e*.*g*., elongated objects, overexposure or discretization artifacts). To eliminate this behavior, we used the ≤-operator instead of the <-operator to additionally detect non-strict local extrema (LoGNSM). However, this increased the amount of false positive detections in background regions and along elongated objects.

Detections in background regions were removed using an intensity threshold (*t*_wmi_) applied to the mean intensity of a small window surrounding the potential detection. The remaining seed points were mostly located properly on the detected objects and remaining false positive detections largely originated from objects that were detected multiple times. To combine redundant objects to a single object, a fusion approach based on hierarchical clustering was used (LoGNSM+F). The hierarchical cluster tree was computed using Ward’s minimum variance method to compute distances between clusters, *i*.*e*., the within-cluster variance was minimized to obtain equally sized clusters [40]. Final clustering was obtained from the complete cluster tree using a distance-based cutoff *t*_dbc_ that was set to the smallest expected object radius *r*_min_, to fuse close redundant detections and to prevent fusion of neighboring objects. A single detection per object was obtained by averaging the feature vectors of all detected seeds in a cluster. In S6 Fig, a screenshot of the graphical user interface used for semi-automatic parameter optimization is shown. Furthermore, S7 Fig shows different parameter settings of the *t*_wmi_ threshold parameter and illustrates how an optimal parameter value can be visually determined.

**Fig 4:**
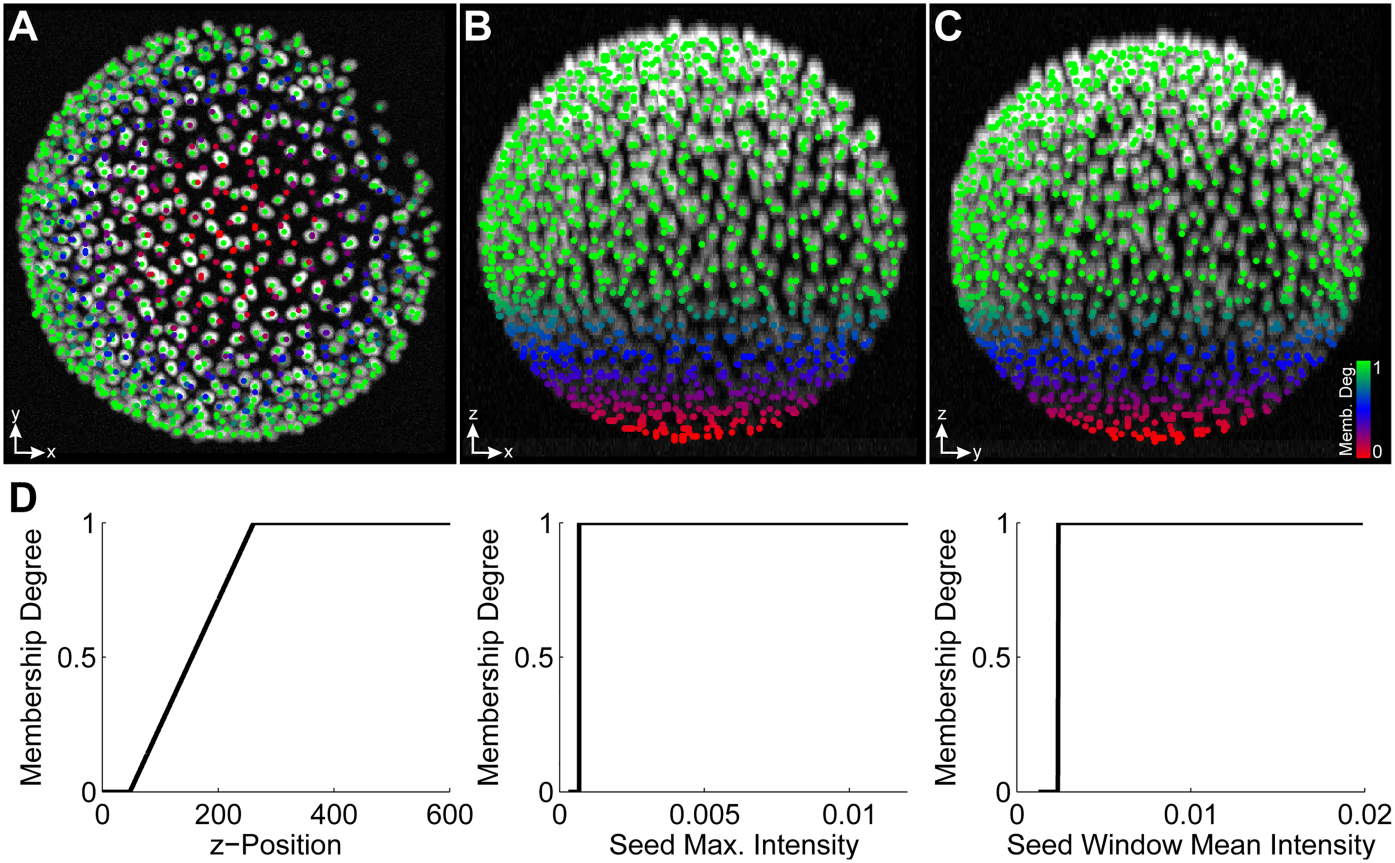
Maximum intensity projection of a 3D benchmark image along the Z, Y and X axis superimposed with detected seed points (A, B, C). Seed points are colored according to their FSMD to the class of a correct detection ranging from red over blue to green for low, medium and high membership degree, respectively. The fuzzy sets used for the individual features are depicted in (D) and the min-operator was used as a fuzzy conjunction to obtain the final membership degree. The uncertainty gradient along the z-axis was introduced due to the signal attenuation at locations farther away from the detection objective and was used in later steps to resolve multiview fusion ambiguities.

As the seed detection stage usually represents one of the first analysis steps, no preceding uncertainty information was considered. To inform down-stream operators about the expected result quality, the uncertainty of the detected seed points was estimated using the window mean intensity, the maximum seed intensity and the z-position features of the extracted objects (LoGNSM+F+U). Besides discarding obvious false positive detections in background regions (intensity-based thresholds), the fuzzy sets for the z-position were adjusted such that seed detections in low contrast regions (farther away from the detection objective) had lower membership degrees to the class of correct objects than objects in the high contrast regions (closer to the detection objective). The final fuzzy set membership degree of an object to the fuzzy set of correct objects was determined using the minimum of the obtained membership degrees and was appended as a new feature to the output matrix of the seed detection algorithm. To filter false positives we set the forward threshold slightly above zero to *α*_11_ = 0.0001 (forward threshold for Operator 1 and Linguistic Term 1). Color-coded visualizations of the detected seed points and the fuzzy sets for the different features are shown in Fig 4.

#### Segmentation

After the seed detection stage, a segmentation operator was used to extract the regions and regional properties of all detected objects from the simulated 3D image stacks. For demonstration purposes, we further improved an algorithm based on adaptive thresholding using Otsu’s method [41] and a watershed-based splitting of merged objects [42] (OTSUWW) as described in [39]. This approach used propagated information from the seed detection stage and estimated FSMD values of extracted segments to improve the algorithmic efficiency and the segmentation quality (OTSUWW+U).

Based on the extracted statistical quantities of the benchmark images (Table 2), we derived the parameter vector ***θ*** = (*a*, *b*, *c*, *d*)^⊤^ of the trapezoidal fuzzy set membership function for each considered feature using the minimum and maximum values as *a*, *d* parameters, respectively. The remaining parameters *b*, *c* were set to the 5%-quantile and the 95%-quantile. This parameterization ensured that all values smaller or larger than the maximum values obtained a membership degree of zero and that 90% of the data range was assigned a membership value of one. Of course, this parameterization is application and data dependent and can be customized, *e*.*g*., to adjust the behavior for extrema at the lower and upper spectrum of the value range. For simplicity, the focus was put on volume and size information of the objects. In the absence of ground truth data, the transition regions for the fuzzy sets can be identified by a manual analysis of objects that deviate from the expectation at the lower and the upper feature value range, *e*.*g*., using software tools such as Fiji, ICY or Vaa3D [43–45]. Alternatively, simple graphical user interfaces that allow interactive post-corrections and parameter adjustments for specific algorithms can be implemented as shown in the example of seed detection in S6 Fig and as discussed in [46, 47].

**Table 2:** Statistical quantities of the benchmark data set.

Feature	Min	Max	Mean	Std.	Med.	5% qt.	95% qt.
**Volume**	449	2016	993.6	247.2	990	617	1405
**Width**	13	31	19.9	2.6	20	15	24
**Height**	13	34	19.9	2.5	20	15	24
**Depth**	3	11	6.2	1.0	6	5	8

Minimum, maximum and quantile values were used to formulate fuzzy sets for each of the features. Individual fuzzy sets were combined using the minimum operator, to obtain a single membership degree value to the fuzzy set of being a valid object.

**Fig 5:**
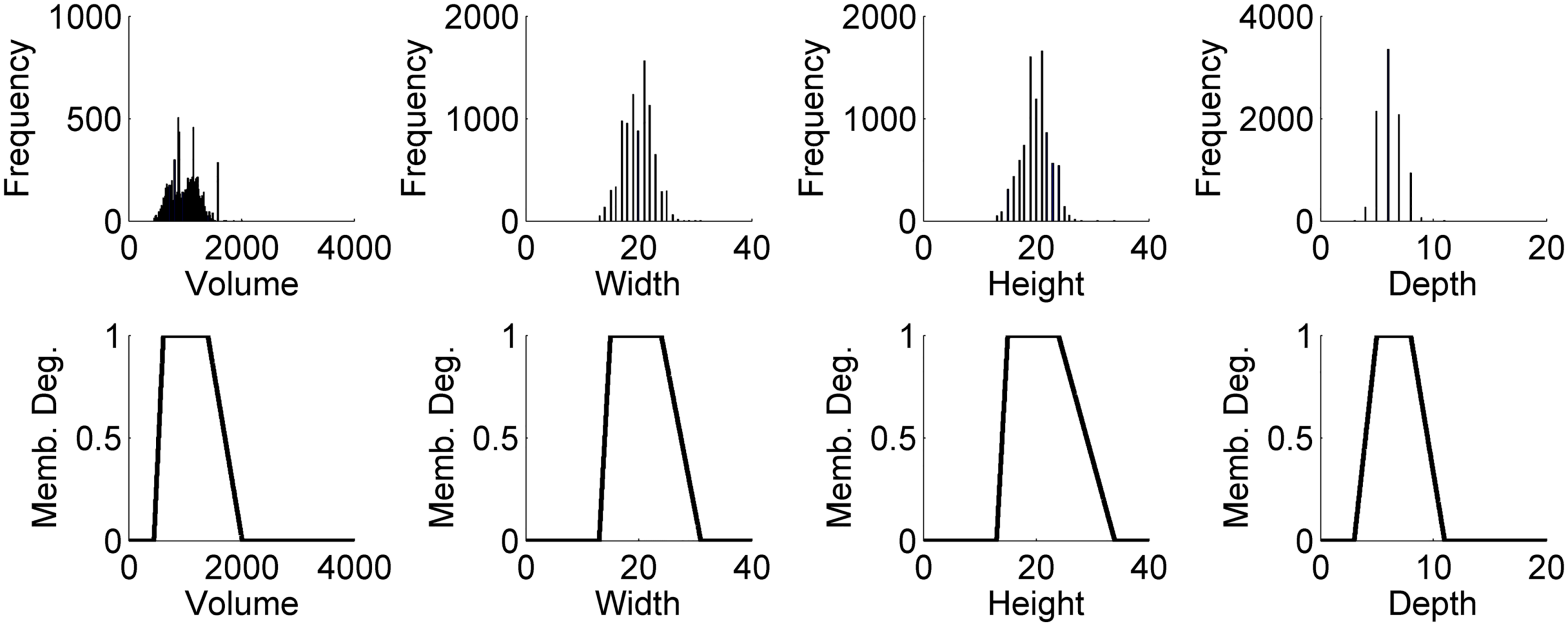
Feature histograms and derived fuzzy sets. Feature histograms (top) and fuzzy set membership functions (bottom) for volume (***θ***_vol_ = (449, 617, 1405, 2016)^⊤^), width (***θ***_w_ = (13, 15, 24, 31)^⊤^), height (***θ***_h_ = (13, 15, 24, 34)^⊤^) and depth (***θ***_w_ = (3, 5, 8, 11)^⊤^).

As depicted in Fig 5, the shapes of the fuzzy set membership functions derived from the statistical quantities resemble the respective distribution observed in the feature histograms. We used the min-operator to combine the individual fuzzy set membership degrees to a single value, *i*.*e*., the combined FSMD value directly corresponded to the membership degree of the feature that deviated the most from the specified expected range (see S1 Note). Of course, the size criteria discussed here should only be considered as an exemplary illustration. There are various other features that can potentially be used to assess and improve segmentation results, *e*.*g*. integrated intensity, edge information, local entropy, local signal-to-noise ratios (SNR), principal components, weighted centroids and many more. Furthermore, if colocalized channels are investigated, complementary information can be used to formulate more complex decision rules. In the case of a fluorescently labeled nuclei and membranes that are imaged in different channels [3, 5, 48], rules like “*each cell has exactly one nucleus*” can be formalized in the same way using the fuzzy set membership functions for a quantification of the available prior knowledge.

The identified FSMD values of segmented objects were then used to detect under-segmentation errors produced by Otsu’s method that needed to be further split to match the expected object size. The OTSUWW+U implementation used the seed points of the LoGNSM+F+U method to correct the under-segmentation errors of the original Otsu segmentation. In contrast to the splitting approach described in [39, 42] (OTSUWW), the uncertainty-guided watershed splitting technique was only applied to objects that were known to be larger than expected. Using a parallelization strategy similar to the one discussed in [39], all segments with combined FSMD values below *β*_21_ (backward threshold for Operator 2 and Linguistic Term 1) that corresponded to objects larger than expected (Case 3, see Methods) were distributed among the available CPU cores and a seeded watershed approach was used for object splitting in each of the cropped regions of the image [42]. This approach was much faster than directly applying the watershed algorithm on the entire image (OTSUWW), due to the uncertainty-guided, locally applied processing of erroneous objects. After splitting merged objects, the connected components of the image were re-identified and the uncertainty values were re-evaluated. This updated information was then provided to the subsequent processing operators.

To further improve the results with respect to false positive detections observed for higher noise levels, segments with combined FSMD values below *α*_21_ = 0.1 (forward threshold for Operator 2 and Linguistic Term 1), *i*.*e*., objects that were smaller than the expected object size (Case 2 and Case 4, see Methods) were removed from the label images. To facilitate the implementation of the uncertainty-guided segmentation, a single fuzzy set for the correct class of objects (Case 1, see Methods) was used to identify objects that needed further consideration. To determine if objects with low FSMD values were smaller or larger than the expectation, a comparison to the boundaries of the expected valid range was performed (trapezoidal fuzzy set parameters *b*, *c*). Threshold values were identified using the interactive graphical user interface presented in [48].

#### Tracking

The final step of the image analysis pipeline was the tracking of all detected objects in order to identify the correct correspondences of detected objects over time in all acquired images. For illustration purposes, a straightforward nearest-neighbor tracking approach implemented in the open-source MATLAB toolbox SciXMiner was used [35, 49]. Each object present in a frame was associated with the spatially closest object in the subsequent frame. This procedure was applied to every frame of the data set in order to obtain a complete linkage of all objects.

In addition to tracking the results of the segmentation methods introduced in the previous section, we tested an alternative approach that combined a flawed segmentation with provided seeds (OTSU+NN+U). Inspired by conservation tracking methods [10], we left the flawed segmentation results produced by the respective algorithms unchanged and provided detected seed points to the tracking algorithm instead of actually using the seed points for splitting in the image domain [31]. As shown in the results section, the watershed-based object splitting was essential to improve the segmentation quality achieved by OTSU. Nevertheless, the tracking algorithm could be extended to decide what information was reliable and suitable for tracking; and it could optionally fall back to the provided seed points if the segmentation quality was insufficient. Therefore, we used the FSMD values provided by the segmentation stage. Using an empirically determined threshold of *β*_31_ = 0.9 (backward threshold for Operator 3 and Linguistic Term 1), all objects with an aggregated FSMD value lower than the threshold were not tracked with the actual segment, but with the seed points contained in the respective segment. The forward threshold parameter was set to *α*_31_ = 0.0 in order to report all tracking results.

## Results

The functionality of the proposed approaches was validated on simulated 3D benchmark images. The data sets contained images with different numbers of objects (SBDE1), different noise levels (SBDE2 and SBDE3) and a set of 50 sequential time points with 1000 moving and interacting objects (SBDE4). The SBDE4 data set included a multiview simulation, *i*.*e*., at each time point, two simultaneous images from opposite direction were generated. An overview of the generated benchmark data sets is provided in S2 Table and a brief description of the data set generation using our XPIWIT software tool [50] is provided in S2 Note and in S1 and S2 Figs.

### Seed point detection validation

To validate the proposed improvements of the LoG-based seed detection algorithm, the SBDE1, SBDE2 and SBDE3 benchmark data sets were used (S2 Table) with the parameters listed in S3 Table and the performance measures described in S3 Note. The obtained values are summarized in Table 3, where each entry of the table corresponds to the arithmetic mean value of the independently obtained results of the ten benchmark images of SBDE1.

**Table 3:** Quantitative assessment of the seed detection performance.

Method	TP	FP	FN	Recall	Precision	F-Score	Distance	Time (s)	KVox./s
**LoGSM**	681.1	**3.3**	202.8	0.77	**1.00**	0.87	1.60	**7.23**	**7259.82**
**LoGNSM**	**813.7**	160.0	**70.2**	**0.91**	0.84	0.87	1.64	7.66	6858.39
**LoGNSM+F**	811.7	4.5	72.2	**0.91**	0.99	**0.95**	**1.59**	7.70	6819.58
**LoGNSM+F+U**	812.5	4.3	71.4	**0.91**	0.99	**0.95**	**1.59**	9.77	6170.05

Quantitative performance assessment of the LoG-based seed detection methods. The criteria are true positives (TP), false positives (FP), false negatives (FN), recall, precision, F-Score, the distance to the reference (Dist., smaller values are better) as well as the achieved time performance measures in seconds (smaller values are better) and voxels per second (larger values are better). All values represent the arithmetic mean of the individually processed benchmark images.

Quantitative analysis confirmed that the proposed extensions of LoGSM could improve the algorithmic performance by up to 9.2% with respect to the F-Score. Although LoGSM had few false positive detections, it missed many objects due to the strict maximum detection (recall of 0.77 and precision of 1.0). The recall was improved by 18.2% to a value of 0.91 by additionally allowing non-strict maxima (LoGNSM). However, this adaption concurrently raised the number of false positives and thus lowered the precision by 16.0% to 0.84, as objects with maximum plateaus were detected multiple times. These multi-detection errors were successfully removed using the proposed fusion technique, which was reflected in an F-Score value of 0.95 for LoGNSM+F(+U), *i*.*e*., compared to the LoGSM method, the F-Score was increased by 9.2%. With regard to processing times, the additional effort required for redundant detection was almost negligible, as the non-strict maximum detection simply detected more seed points during the same iteration over the image. The seed point fusion was performed directly in the feature space and was therefore also insignificant compared to the preceding processing steps. For the feature set described here, using the uncertainty-based object rejection (LoGNSM+F+U) only slightly improved the results compared to directly fusing and filtering the data using the hard intensity threshold and increased the processing time by 35%. LoGNSM+F yielded almost identical results and required only 6% more processing time compared to LoGSM. Nevertheless, all objects were equipped with an uncertainty value that was propagated through the pipeline and proved to be beneficial to filter, fuse and correct the extracted data in subsequent steps. In addition, it should be noted that the processing time required for the analysis of larger images easily exceeds that of fuzzy set calculations.

**Fig 6:**
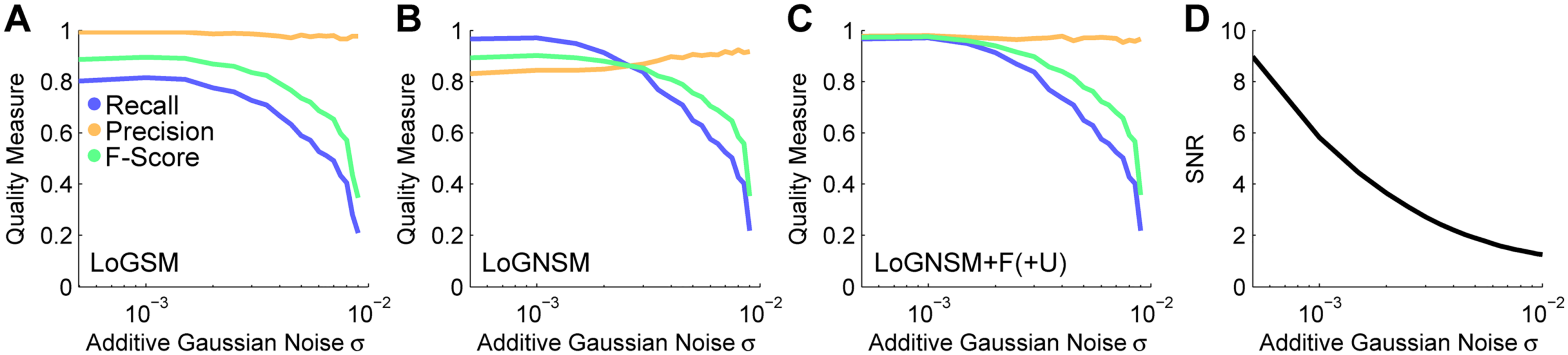
Assessment of the seed detection performance for the different levels of additive Gaussian noise contained in the SBDE2 data set. The performance measures recall, precision and F-Score are plotted versus the additive Gaussian noise level parameter *σ*_agn_ for LoGSM (A), LoGNSM (B) and LoGNSM+F(+U) (C). As LoGNSM+F and LoGNSM+F+U produced identical results with respect to recall, precision and F-Score, the plots are combined to a single panel (Table 3). The influence of the noise level on the signal-to-noise ratio of the images is plotted in (D). Optimal thresholds for the *t*_wmi_ parameter were identified using an interactive graphical user interface depicted in S6 Fig. Furthermore, examples for different parameter settings and the identified optimal parameters are shown in S7 Fig.

Furthermore, we tested seed detection performance under different image noise conditions using the SBDE2 data set, which contained images with different settings for the additive Gaussian noise standard deviation (*σ*_agn_ ∈ [0.0005, 0.01]). Seeds from these images were extracted using the LoGSM, LoGNSM, LoGNSM+F and LoGNSM+F+U algorithms and the intensity thresholds were determined for each of the noise levels individually using the semi-automatic graphical user interface described in [47]. For higher noise levels, the number of detections in background regions increased and detections in low contrast regions became ambiguous. The manual threshold was therefore adjusted such that the false positive detections were minimized and only unambiguous seeds were considered. This continuous threshold adaptation was the reason for constant (Fig 6A and 6C) or increasing precision (Fig 6B) in noisy image regions, because false positives were easier to identify than false negative detections. Objects were robustly detected down to a signal-to-noise ratio of 5 (Fig 6), which was close to the visual limit of detection [51] and emphasized the uncertainty-based improvements. The same analysis was performed on a data set with different levels of Poisson noise (SBDE3) and the results are shown in S3 Fig.

### Segmentation validation

The segmentation performance was validated using the SBDE1, SBDE2 and the SBDE3 data sets (S2 Table). In addition to the algorithms OTSU, OTSUWW, OTSUWW+U, the segmentation quality obtained by the TWANG method (see [39] for details) was added and quantitatively compared to a TWANG version (TWANG+U) that relied on the improved seed detection operator introduced in the previous section (LoGNSM+F+U). The respective parameterization and a brief description of each algorithm is provided in S4 Table) and the validation measures are summarized in S3 Note and [52]. In Table 4, the quantitative segmentation quality results obtained on the SBDE1 data set are summarized. The Rand index (RI) value was almost identical for all algorithms and OTSU yielded the highest value. The enhanced adaptive threshold-based techniques yielded an 11.0% better Jaccard index (JI) value than TWANG+U and the best normalized sum of distances (NSD) value was obtained by OTSUWW+U. Considering the results for RI, JI and NSD, the global threshold-based techniques (OTSU*) produced slightly more accurate results (0.3%, 5.3% and 12.0%, respectively) for the objects they resolved compared to the best results of the TWANG-based methods. However, both TWANG-based methods produced the minimal amount of topological errors with respect to split and merged objects compared to all OTSU-based methods. The number of added objects was minimal for OTSUWW+U. TWANG+U produced slightly more added objects than TWANG but efficiently detected far more objects. Thus, the F-Score values achieved by TWANG+U were further increased by 8.4% compared to TWANG, *i*.*e*., TWANG+U produced the best results with the fewest topological errors (F-Score 0.9). The low amount of split and merged nuclei for TWANG originated from the single-cell extraction strategy that was used instead of a global threshold as performed in the OTSU-based methods. The relatively large amount of added objects detected by the TWANG segmentation were mostly not real false positive detections, but segments with more than 50% of the voxels intersecting with the image background instead of the actual object. Thus, these detections were erroneously considered as false positives.

**Table 4:** Quantitative assessment of the segmentation performance.

Method	RI	JI	NSD (×10)	HM	Split	Merged	Added	Missing	Rec.	Prec.	F-Score	Time (s)	KVoxel/s
**OTSU**	**97.92**	27.82	3.77	6.67	25.00	370.50	42.40	264.00	0.29	0.78	0.42	**6.11**	**8589.46**
**OTSUWW**	97.91	**27.99**	3.29	6.48	88.60	96.30	57.90	276.20	0.58	0.78	0.67	42.41	1237.72
**OTSUWW+U**	97.91	27.98	**3.15**	5.61	50.60	57.80	**13.30**	268.40	0.64	**0.90**	0.75	26.40	2035.66
**TWANG**	97.67	26.59	3.58	**4.62**	**0.00**	**6.70**	75.80	193.50	0.77	**0.90**	0.83	11.02	4774.83
**TWANG+U**	97.81	25.30	3.64	4.75	**0.00**	12.40	103.70	**59.50**	**0.91**	0.89	**0.90**	11.15	4728.84

The criteria used to compare the algorithms are the Rand index (RI), the Jaccard index (JI), the normalized sum of distances (NSD) and the Hausdorff metric (HM) as described in [52]. Additionally, the topological errors were assessed by counting split, merged, added and missing objects. Precision, recall and F-Score are based on the topological errors by considering split and added nuclei as false positives and merged and missing objects as false negatives, respectively. The achieved time performance was measured in seconds (smaller values are better) and voxels per second (larger values are better). All values represent the arithmetic mean of the individually processed benchmark images.

With regard to processing times OTSU was the fastest approach; however, it was not really an option for a reliable analysis of the image data, because without the uncertainty-based extension the segmentation results were of poor quality. TWANG and TWANG+U were 1.8 times slower than the plain OTSU method but 2.4 and 3.8 times faster than OTSUWW and OTSUWW+U, respectively. TWANG+U was the most precise approach (F-Score 0.9). Furthermore, OTSUWW+U was 1.6 times faster than OTSUWW due to the focused object splitting and produced better results due to the improved seed detection and noise reduction.

These results confirmed that uncertainty information can be efficiently exploited to guide computationally demanding processing operators to specific locations, thereby speeding up processing operations while preserving or even improving result quality.

**Fig 7:**
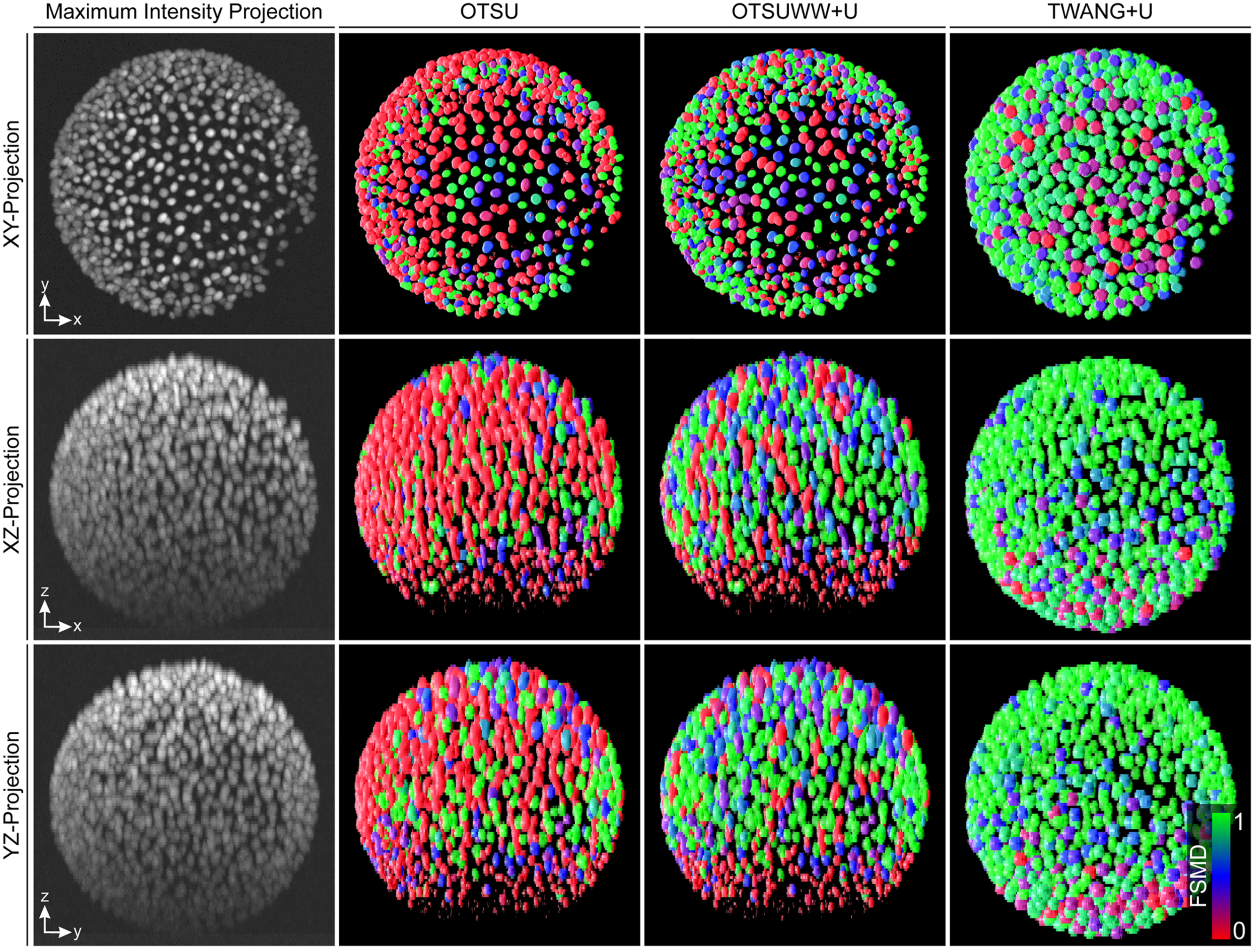
Maximum intensity projections of the raw image and exemplary volume renderings of the automatic segmentation results produced by OTSU, OTSUWW+U and TWANG+U from different viewpoints (XY, XZ and YZ). The FSMD of individual detected objects was estimated using the morphological criteria volume and size and was used for coloring (red over blue to green for low, medium and high membership degree, respectively).

Exemplary FSMDs of the final segmentation results of different algorithms are depicted in Fig 7. These visualizations allow assessment of segmentation quality and the identification of potential problems even by non-experts. All algorithms suffered from light attenuation in the axial direction. In particular, the techniques that relied on a single global intensity threshold had problems identifying the objects located in these low-contrast regions. Besides missing many objects, OTSU also merged many of the high intensity objects into a single large blob. Due to lower sampling in the z-direction, many mergers occurred in this direction. However, these merged regions could, to a large extent, be successfully split using the proposed seed-based splitting techniques (OTSUWW, OTSUWW+U). As TWANG directly operated on the provided seeds, it was still able to extract most of the objects in these regions and yielded even higher recall values using the LoGNSM+F+U seed points. However, due to the low-contrast, the segmentation quality of the extracted segments in these regions was reduced. In Table 3, this is reflected by the increased number of added objects for TWANG and TWANG+U, which were mostly no real false positives as described above.

**Fig 8:**
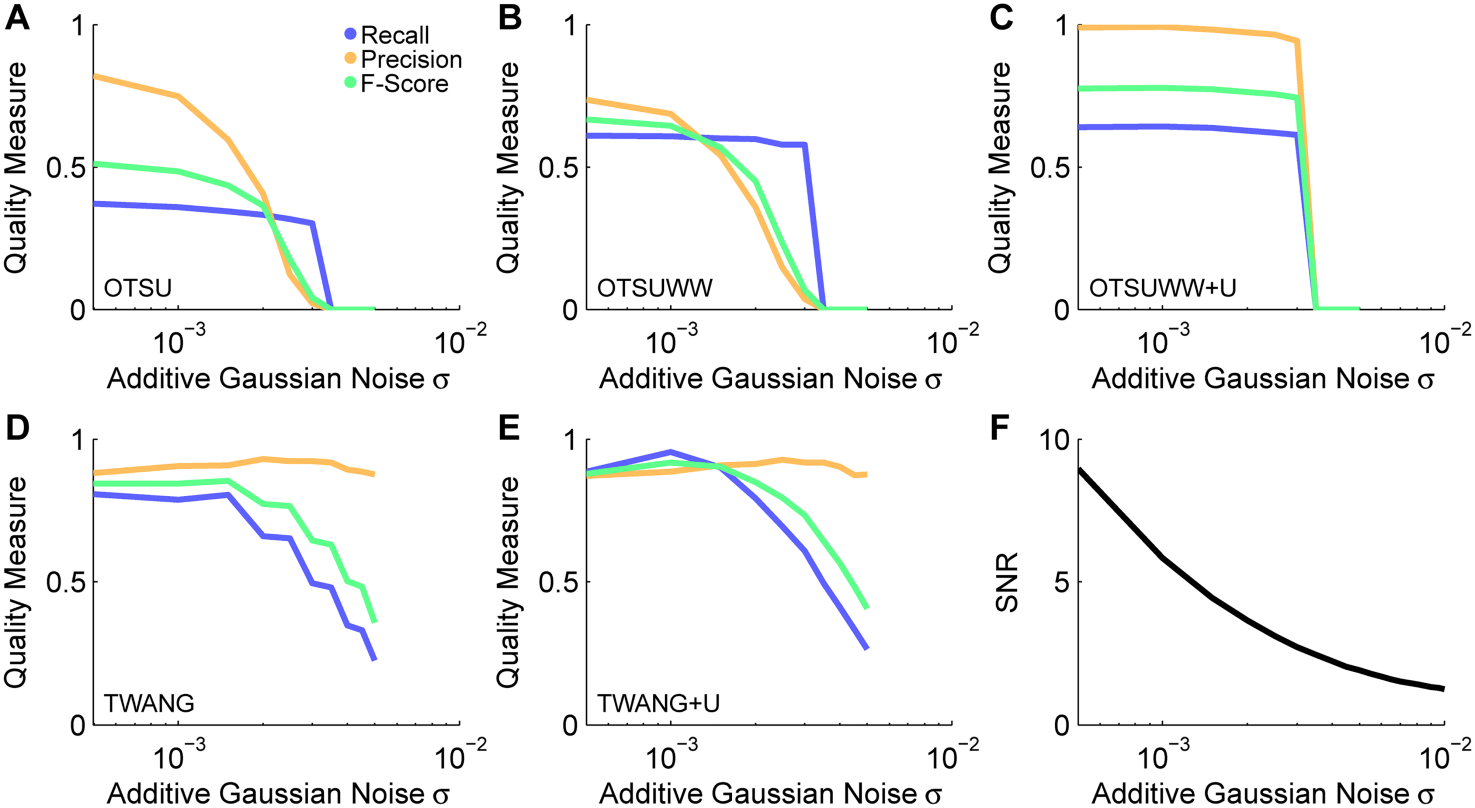
Performance evaluation of the segmentation methods OTSU (A), OTSUWW (B), OTSUWW+U (C), TWANG (D) and TWANG+U (E) on images of the SBDE2 data set with different signal-to-noise ratios. The methods based on adaptive thresholding (OTSU, OTSUWW) suffered from high noise levels and produced a successively increased amount of false positive detections, which could be efficiently suppressed using the uncertainty framework-based extension (OTSUWW+U). The result quality of both TWANG versions directly correlated with the quality of the provided seed points, *i*.*e*., TWANG+U benefited from the improved detection rate of LoGNSM+F+U.

To investigate the impact of the signal-to-noise ratio of the images on the segmentation quality, the benchmark data set SBDE2 was processed using all five algorithms. The segmentation quality of all adaptive thresholding-based methods was heavily affected by the noise level of the images yielding poor precision and recall values even for the lowest noise levels (Fig 8A and 8B). This was caused by the global threshold, which merged a lot of objects and detected a high amount of false positive segments. The uncertainty-based method OTSUWW+U successfully preserved the increased recall of OTSUWW and at the same time substantially increased the precision to an almost perfect level for noise parameters of *σ*_agn_ < 0.003 (Fig 8C). The increasing number of small segments observed for OTSU and OTSUWW could be efficiently filtered using the uncertainty-based object rejection. As TWANG heavily depended on the quality of the provided seeds, the observed curves in Fig 8C and 8D show a high correlation to seed detection performance (Fig 6A and 6C) and render it as a suitable method even for higher noise levels. The improved seed detection of LoGNSM+F+U also directly affected the quality of the TWANG+U segmentation. Note that the seed points were adjusted for each of the noise levels, *i*.*e*., subtle variations in the precision and recall (Fig 8D and 8E) values were caused by a subjective manual threshold adaptation. Qualitatively, the same algorithmic behavior was observed on the Poisson noise flawed image material (SBDE3) and the results obtained on this benchmark data set are shown in S4 and S5 Figs.

### Tracking validation

**Fig 9:**
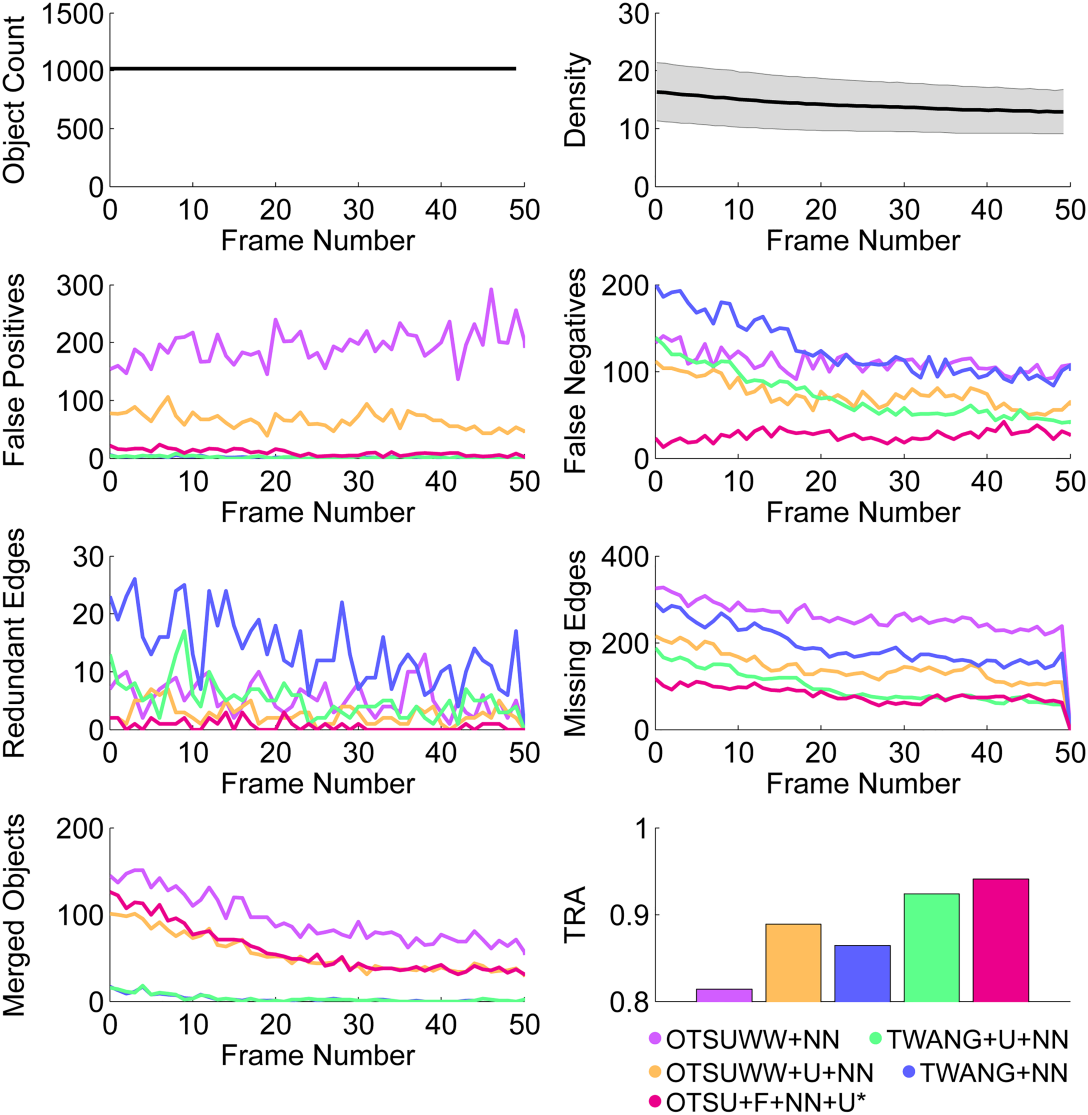
Quantitative performance assessment of a nearest neighbor tracking algorithm (NN) applied on different segmentation results obtained on the SBDE4 data set. Two algorithms without uncertainty-based improvements as described in [39] (OTSUWW, TWANG) were compared to enhanced pipelines that explicitly incorporate prior knowledge-based uncertainty treatment (OTSUWW+U, TWANG+U, OTSU+F+NN+U). (*) indicates that the respective algorithm did not produce a usable segmentation image, as the correction was solely performed at the tracking step.

The tracking validation was performed on the SBDE4 data set, which consisted of 50 frames with two simultaneously acquired rotation images for each frame, yielding a total number of 100 frames that needed to be processed. Segmentation was performed using OTSUWW, OTSUWW+U, TWANG and TWANG+U separately for all time points and view angles. To obtain a single set of objects for each time point, segmentation results of different view angles were fused using the segment-based fusion approach described in S4 Note. The centroids of all detected objects were then used to perform the nearest neighbor tracking (NN). In addition, OTSU+F+NN+U was applied to the test data set, *i*.*e*., the Otsu-based threshold was applied to both rotation images independently and the resulting binary images were then fused using the maximum pixel value of the two images. FSMD values were then estimated on the connected components of the fused image using the same fuzzy sets that were used in the segmentation step. The obtained tracking results are summarized in Table 5 and Fig 9.

**Table 5:** Quantitative assessment of the tracking performance.

Method	TP	FP	FN	Redundant	Missing	Merged	Rec.	Prec.	F-Score	TRA	Time (s)	KVoxel/s
**OTSUWW+NN**	905.33	194.10	110.67	5.51	256.88	94.33	0.89	0.82	0.86	0.81	51.36	1020.81
**OTSUWW+U+NN**	941.94	65.65	74.06	2.63	141.39	55.04	0.93	0.93	0.93	0.89	33.68	1556.68
**TWANG+NN**	889.73	**1.73**	126.27	13.75	191.16	3.29	0.88	**1.00**	0.93	0.86	**14.27**	**3674.06**
**TWANG+U+NN**	943.90	1.76	72.10	5.16	96.55	**3.27**	0.93	**1.00**	0.96	0.92	21.55	2432.89
OTSU+F+NN+U*	**989.63**	9.84	**26.37**	**0.67**	**79.67**	59.33	**0.97**	0.99	**0.98**	**0.94**	18.66	2809.72

Quantitative performance assessment of a nearest neighbor tracking algorithm (NN) applied on different segmentation results. Two algorithms without uncertainty-based improvements (OTSUWW, TWANG) were compared to enhanced pipelines that explicitly incorporated prior knowledge-based uncertainty treatment (OTSUWW+U, TWANG+U). Furthermore, an OTSU-based segmentation with additional seed points from LoGNSM+F+U (OTSU+F+NN+U) was used with an adapted tracking algorithm. Note that the segmentation produced by OTSU+F+NN+U was not usable for other purposes than tracking due to many merged regions (indicated by (*)). The validation measures correspond to true positives (TP), false positives (FP), false negatives (FN), redundant edges (Red.), missing edges (Miss.) and merged objects (Merg.). Furthermore, recall, precision, F-Score and the TRA measure were calculated as described in S3 Note. Processing times are average values for applying segmentation and tracking on a single image and were measured in seconds (lower values are better) and voxels per second (higher values are better).

Without uncertainty treatment, both pipelines reached the lowest tracking accuracy with respect to the tracking quality measure (TRA, see S3 Note) due to an increased number of missing objects (recall of 0.89 for OTSUWW+NN, and 0.88 for TWANG+NN) and a high number of false positive detections (precision of 0.82 for OTSUWW+NN). Of course, these missing objects directly correlated with the number of missing edges and explain the 222.4% (OTSUWW+NN) and 139.9% (TWANG+NN) higher amount of missing edges compared to the best scoring algorithm in this category (OTSU+F+NN+U). Furthermore, OTSUWW+NN suffered from many merged regions, which contributed to an 8.2% lower recall value than OTSU+F+NN+U. In contrast to this result, all uncertainty-enhanced methods provided comparable results, with the best results achieved by OTSU+F+NN+U and TWANG+U+NN. Although the amount of false negatives of TWANG+U+NN was almost half that of TWANG+NN, the detection rate was still the largest problem of this pipeline resulting in a 21.2% higher amount of missing edges compared to OTSU+F+NN+U. However, OTSU+F+NN+U still suffered from the same under-segmentation tendency as observed for OTSUWW+NN and OTSUWW+U+NN; consequently, missing edges were its main problem. With respect to false positive detections both TWANG-based methods provided the best results with precision values of 1.0, because simultaneous multiview acquisition enabled high quality seed detection. This reflects an improvement of the precision obtained by the TWANG-based methods of 22.0% compared to OTSUWW+NN, which did not have an uncertainty-based object exclusion and thus an increased amount of false positive detections in background regions. The reason for the slightly higher number of redundant edges observed for the two TWANG-based methods is not yet fully clear. Most likely, the seed detection already provided a redundant seed to the segmentation method, which produced two nearby segments that were in turn counted as a redundant segment by the tracking evaluation. However, even for the worst algorithm in this category (TWANG+NN) redundant edges were only observed for 1.4% of the tracked objects and thus play a minor role compared to the other tracking errors. As shown in Fig 9, the number of false negatives and merged objects directly correlated with the density of the objects, *i*.*e*., the closer the objects were to each other, the more under-segmentation errors occurred. However, this was not the case for TWANG-based methods due to the explicit prior knowledge about the object size that was incorporated to the algorithm. The OTSU+F+NN+U method produced the best tracking result and was the second fastest method (TRA value of 0.94 and average processing time of 18.7*s*) closely followed by TWANG+U+NN (TRA value of 0.92 and average processing time of 21.6*s*). Compared to OTSUWW+NN, TWANG+U+NN and OTSU+F+NN+U provide superior quality in all categories and in particular an increase of the TRA measure by 13.6% and 16.0%, and a decrease of processing times by 58.0% and 63.7%, respectively. Thus, the two latter methods represent the best quality vs. speed trade-off and are suitable for large-scale analyses. Although OTSU+F+NN+U provided excellent results in this comparison, it should be noted that the extracted segmentation masks were largely merged and an object splitting approach as performed for OTSUWW would be required if object properties need to be known. However, an additional object splitting approach would eradicate the performance benefit of the method and pipelines such as TWANG+U+NN pipeline should be favored in this case.

Falling back on seed point information was not beneficial for segmentation methods like TWANG, where the algorithmic design already only extracts a single segment per seed point and literally no merged objects exist. Future work could potentially address a combination of the LoGNSM+F+U and the OTSU-based segmentation for seed detection and to feed these seeds to the TWANG algorithm, to reach both a further reduced amount of missed objects and a reduced amount of merged objects. Moreover, the temporal coherence was not yet considered in the investigated framework, *i*.*e*., additionally allowing a nearest neighbor matching over multiple frames could potentially also help to reduce the number of missing and redundant detections.

### Application to light-sheet microscopy images of zebrafish embryos

**Fig 10:**
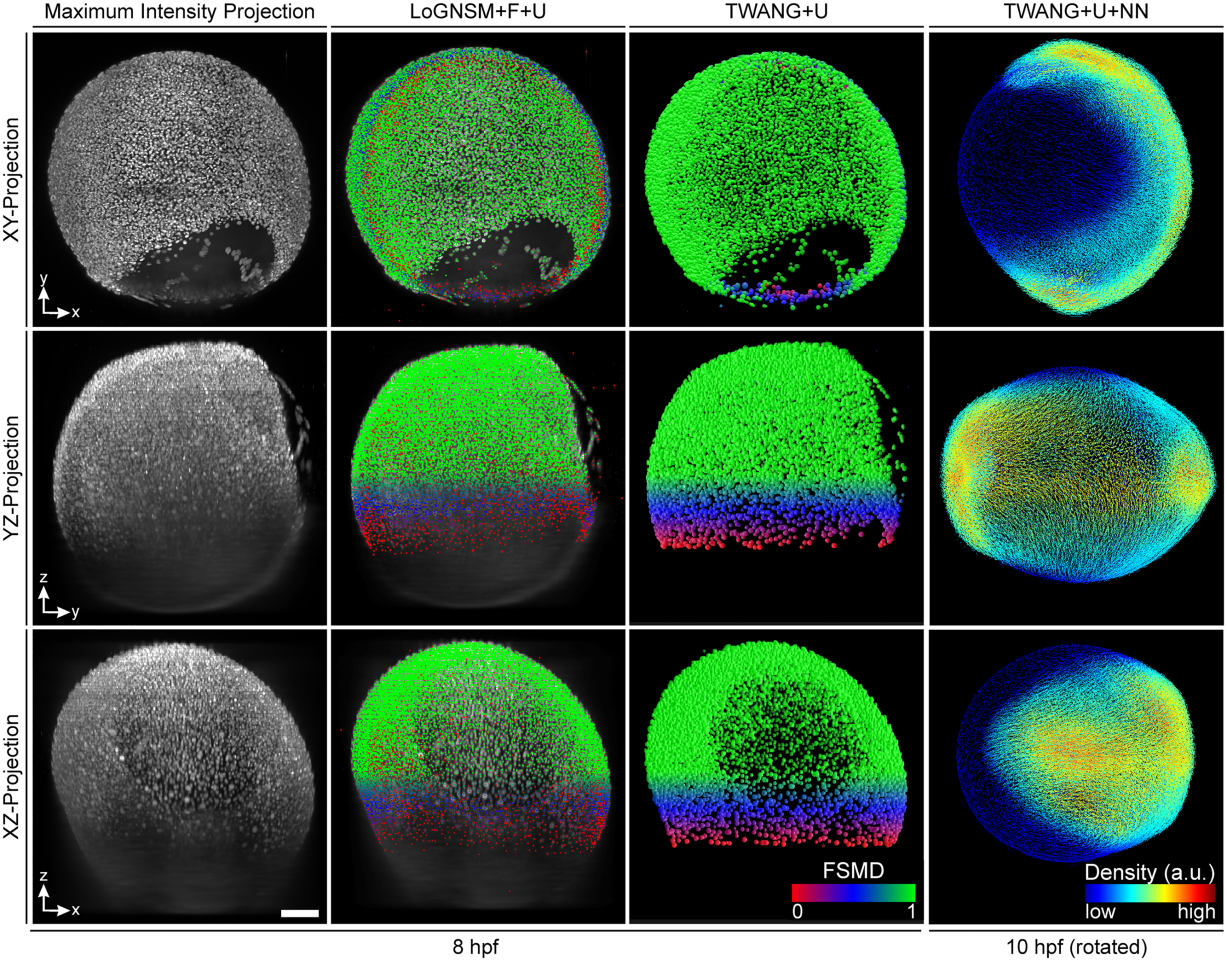
Application of the presented framework to 3D+t light-sheet microscopy images of zebrafish embryos. The panels show the maximum intensity projections on the XY, YZ and XZ planes. Seed points were extracted using the LoGNSM+F+U method and the improved seed points were used for segmentation using the TWANG+U method. Extracted objects of two opposite views were combined using a segment-based fusion approach (S4 Note) and the fused object locations were then used for tracking (TWANG+U+NN). The first three columns show results of a single view at approximately 8 hours post fertilization (hpf) with the color-code according to the FSMD values of the extracted objects (red over blue to green for low, medium and high FSMD values). The last column shows the results after multiview fusion and tracking at about 10 hpf using a cell density for coloring. In addition, the embryo was oriented along the coordinate axes, such that the anteroposterior axis was aligned with the Y axis (animal pole on the top) and the dorsoventral axis aligned with the X axis (dorsal to the right) [37]. Data were taken from our previously published work [37–39, 47]. Scale bar, 100 *μ*m.

The presented framework was successfully used for the automated analysis of large-scale 3D+t microscopy images of developing zebrafish embryos [37–39, 47]. In particular, we used the LoGNSM+U method, *i*.*e*., seed points were detected using a non-strict local maximum detection with a subsequent fusion of redundant detections and a false positive suppression based on the axial location of the seeds as well as their fluorescence intensity information. These seeds were then provided to the TWANG algorithm as described in [39] and the segments of different views were combined using a segment-based fusion approach (S4 Note and [37]). Finally, a nearest-neighbor tracking was applied to the detected objects to obtain the movement trajectories (TWANG+U+NN). As there were about 8 million dynamically interacting objects in total for the investigated time period of about 3–10 hours post fertilization (hpf), manual labeling and validation were impossible. Qualitative results of seed detection, segmentation and the tracking stage are depicted in Fig 10 and movement dynamics are shown in S3 Video. The OTSU-based approaches were not suitable for processing these images and led to a large amount of missed detections and under-segmentation errors. This was mainly caused by intensity variations, light scattering and successively decreasing image quality in the axial direction. A direct comparison of the results obtained by TWANG and OTSU are shown in S8 Fig). Moreover, the memory demands of the watershed-based post-processing approaches limited their application to the full-sized 3D images as discussed in [39].

## Conclusions

In this contribution, we presented a general concept for the mathematical formulation of prior knowledge and showed how image analysis pipelines can be equipped with formalized prior knowledge in order to make more elaborate decisions. The framework includes the propagation of estimated result uncertainties, which inform downstream pipeline operators about the validity of their input data and potentially improve their results. Besides these general concepts, we demonstrated how an exemplary pipeline consisting of seed point detection, segmentation, multiview fusion and tracking could be systematically extended by the proposed uncertainty considerations in order to filter, repair and fuse produced data. The performance of all proposed improvements was quantitatively assessed on a new and comprehensive validation benchmark inspired by light-sheet microscopy recordings of live specimen. In comparison to plain pipelines, the extensions demonstrated superior performance compared to the plain pipelines and only had a low impact on the processing times due to the lightweight adaptations of the propagated feature matrices. Thus, the proposed framework represents a powerful approach to improve the quality and efficiency of image analysis pipelines. Several components of the presented framework were successfully used to analyze large-scale 3D+t light-sheet microscopy images of developing zebrafish embryos as described in the previous section and [35, 37].

For simplicity, we primarily focused on simple processing methods to illustrate the general concepts. However, extending more complex seed detection, segmentation or tracking algorithms with the presented concepts of filtering, splitting and fusion should work analogously if the uncertainty-based corrections are considered as a post-processing strategy of each processing operator. The tracking step in particular offers great potential for further improvements. The uncertainty framework could be exploited to classify movement events, *e*.*g*., the detection of object divisions or the reconstruction of missing objects using the temporal coherence of the objects. Moreover, it may be interesting to investigate the influence of the point spread functions onto the quality of image analysis operators and to potentially exploit knowledge about their respective anisotropic shape for uncertainty assessment. However, that was beyond the scope of this paper and will be addressed in upcoming work. In addition to the algorithmic improvements, we showed how the respective fuzzy sets can be parameterized based on available prior knowledge, such as feature histograms or knowledge about acquisition deficiencies. The respective shape of the fuzzy sets had to be determined based on the desired outcome. For instance, the false positive suppression at the seed point detection stage could be performed with a fixed threshold instead of an explicit usage of fuzzy sets for the intensity-based features. However, modeling the increasing uncertainty (decreasing FSMD value) in regions farther away from the detection objective using a trapezoidal shape turned out to be more appropriate. Further work on the automatic determination of the involved fuzzy sets is needed, *e*.*g*., using a semi-automatic approach for a manual classification of a representative subset of data.

## Supporting information

S1 Note Fuzzy conjunction operators. (PDF)

S2 Note Validation benchmark. (PDF)

S3 Note Performance assessment. (PDF)

S4 Note Multiview fusion. (PDF)

S1 Fig Pipeline schematic of the benchmark simulation. (A) Illustration of the embryo simulation. Starting with an initial object, multiple division cycles were simulated including object interaction, object divisions and morphological constraints. Due to inner and outer bounding spheres and the between-object interactions, cells migrated from the animal pole (AP) to the artificial vegetal pole (VP), which produced a behavior similar to the epiboly movement of zebrafish embryogenesis. (B) The performed steps for a realistic simulated 3D+t benchmark. The left column reflects the object simulation and returned raw images that contained dynamic objects and the associated ground truth data. The right column contains the acquisition simulation, which distorted the simulated images by an artificial signal attenuation, a point spread function simulation (PSF), a dark current image simulation, Poisson distributed photon shot noise and additive Gaussian noise. Steps shaded in gray could optionally consider image rotation, if a multiview experiment was simulated and the output operators are indicated by dashed edge lines (adapted from [38]).
(TIF)

S2 Fig Maximum intensity projections of simulated benchmark images. (A) Maximum projections of an extracted division cycle of one simulated nucleus [53]. Time increases from left to right and top to bottom. Single objects were randomly initialized and simulated for a predefined experimental duration. This approach yielded a simulated embryo including object movement, object interaction and object divisions with available ground truth (B). Generated raw image sequences were manipulated to simulate various acquisition conditions, such as different levels of additive Gaussian noise (C). The ground truth enabled a quantitative analysis of the algorithmic performance on realistic image data.
(TIF)

S3 Fig Seed detection performance for LoGSM (A), LoGNSM (B) and LoGNSM+F(+U) (C) on the SBDE3 data set with different levels of Poisson noise. The performance measures recall, precision and F-Score are plotted versus the Poisson noise scale (inversely correlated with the noise level, see [54] for implementation details). The qualitative behavior matches the one observed for additive Gaussian noise. The influence of the Poisson noise scale on the signal-to-noise ratio of the images is visualized in (D). Optimal thresholds for the *t*_wmi_ parameter were identified using the interactive graphical user interface depicted in S6 Fig. Furthermore, examples for different parameter settings and the identified optimal parameters are shown in S7 Fig.
(TIF)

S4 Fig Performance evaluation of the segmentation methods OTSU (A), OTSUWW (B), OTSUWW+U (C), TWANG (D) and TWANG+U (E) on images of the SBDE3 data set with different levels of Poisson noise. The methods based on adaptive thresholding suffered from high noise levels and produced a successively increased amount of false positive detections (OTSU, OTSUWW), which could be efficiently suppressed using the uncertainty framework-based extension (OTSUWW+U). The precision jump at the penultimate noise level observed for OTSUWW+U is actually not an artifact, but caused by a decreased amount of erroneous detections in the lower z-layers that fall below the minimum size criterion due to increased noise-related object splits (see column OTSUWW+U in Fig 7). However, it should be noted that such artificially high noise levels are unlikely to occur for real fluorescence microscopy images of stained cellular nuclei. Analogous to the results obtained for different additive Gaussian noise levels, the result quality of both TWANG versions directly correlated with the quality of the provided seed points, *i*.*e*., TWANG+U benefited from the improved detection rate of LoGNSM+F+U.
(TIF)

S5 Fig Maximum intensity projections of two raw images and exemplary volume renderings of the automatic segmentation results produced by OTSU, OTSUWW, OTSUWW+U, TWANG and TWANG+U for two different Poisson noise levels (signal-to-noise ratio (SNR) of 2.5 and 3.5, respectively). A random color-code was applied to visualize under- and over-segmentation errors. Both OTSU and OTSUWW did not produce satisfactory results with a lot of background noise detections and under-segmentation errors. The uncertainty-based improvements help to improve the quality for OTSUWW+U, and TWANG(+U) performed reasonably well depending on the quality of the provided seeds.
(TIF)

S6 Fig Screenshot of a graphical user interface used for semi-automatic adjustment of the framework parameters. The three main panels depict the maximum intensity projection views of the XY, XZ and YZ plane of an image containing simulated cell nuclei (white objects) with superimposed centroids of detected objects (1). Undesired objects can be filtered based on the feature values, such as spatial constraints or intensity constraints (2). Settings can be propagated to all other images of the project to accelerate the workflow and filtered results can be saved as *.csv files for further processing (3). All parameters used in the uncertainty propagation framework, *i*.*e*., the fuzzy set membership parameterization of any of the available features can be performed in this GUI. The color-code from red (low FSMD) over blue (medium FSMD) to green (high FSMD) provides an instant visual feedback of the membership degree obtained for the individual objects and helps to identify the optimal settings (4).
(TIF)

S7 Fig Results of the LoGNSM+F+(U) seed detection algorithm obtained on two images of different levels of Poisson noise. The columns show the filtered seed points for different settings of the *t*_wmi_ threshold parameter with a too low threshold (left column, many false positive detections), a too high threshold (center column, many false negatives) and a visually determined good threshold (right column, best visually observable balance of false positives and false negatives). For higher noise levels, finding an optimal threshold becomes ambiguous, due to low signal to noise ratio at lower z-slices. Thus, the selection of the threshold largely depends on the respective preference of the user and can cause small fluctuations in the quantitative evaluation for different noise levels (see Figs 6 and 7 in the main text as well as S3 and S5 Figs).
(TIF)

S8 Fig Exemplary comparison of applying OTSU and TWANG to a large-scale light-sheet microscopy image of fluorescently labeled cell nuclei of a zebrafish embryo (single time point at about 9 hours post fertilization). OTSU completely failed to extract meaningful information in this case and only captured a few correct nuclei by chance. TWANG could resolve most of the nuclei correctly and by design largely avoids under-segmentation errors. As the results provided by the plain OTSU algorithm were not properly extracting the embryonic shape, the watershed-based post-processing steps used in OTSUWW and OTSUWW+U were skipped. The results of TWANG+U are visually indiscernible from the plain TWANG results and we thus only show one panel for convenience. Furthermore, due to the image size of about 5 GB per frame, applying the 3D watershed algorithm on the entire image was not possible given the memory limitations of 32 GB on our test computer. A lack of ground truth for these large-scale data sets only allowed a qualitative comparison (see [39] for details). Scale bar: 100*μm*.
(TIF)

S1 Table Nomenclature, abbreviations and symbols used in the present manuscript. (PDF)

S2 Table The benchmark datasets used for the validation experiments. Columns list the dataset identifier (Name), the number of images (Im.), the number of noise levels (N.), the number of objects (Objects), the image resolution, the noise parameter ranges for *σ*_agn_ and the dataset description.
(PDF)

S3 Table Abbreviations, parameterizations and descriptions of the investigated seed detection algorithms. (PDF)

S4 Table Abbreviations, parameters and descriptions of the segmentation algorithms. (PDF)

S1 Video 3D renderings of the simulated benchmark data set used for validation of the presented methods. The left half of the video shows the ground truth labels with a random color code. The right part shows the simulated raw image after small video snippets of simulated cell nuclei were placed at the simulated cell locations.
(MOV)

S2 Video Slices of a simulated benchmark image including the acquisition simulation. In comparison to the raw image shown in S1 Video, image noise, signal attenuation and signal blur can be observed.
(MOV)

S3 Video Tracking results obtained with the described pipeline on 3D+t light-sheet microscopy data of a developing zebrafish embryo for about 3–10 hpf. Seed point detection (LoGNSM+F+U), segmentation (TWANG+U) and segment-based multiview fusion (S4 Note) were used to obtain reliable detections of the fluorescently labeled nuclei for each of the frames. Using a nearest-neighbor tracking algorithm (TWANG+U+NN), trajectories of the individual objects were identified. The embryo was oriented along the coordinate axes, such that the anteroposterior axis was aligned with the Y axis (animal pole on the top) and the dorsoventral axis aligned with the X axis (dorsal to the right) [37]. Data were taken from our previously published work [37–39, 47]. The color-code represents the density of objects ranging from blue over green to red for low, medium and high density, respectively. Cells continuously divide over the course of development and the depicted stages range from a few thousand cells up to more than 20000 cells at the end of the movie [37]. At later stages, convergence and extension movements cause increased cell densities along the prospective anteroposterior axis of the animal.
(MOV)
