# Correction: Fuzzy-based propagation of prior knowledge to improve large-scale image analysis pipelines

**DOI:** 10.1371/journal.pone.0195394

**Published:** 2018-03-29

**Authors:** Johannes Stegmaier, Ralf Mikut

The following information is missing from the Funding section: The Deutsche Forschungsgemeinschaft and the Open Access Publishing Fund of Karlsruhe Institute of Technology funded the open access publishing fee. The funders did not play any role in the study design, experiments performed or interpretation of results presented in the publication.
