# Supplementary material for: Fuzzy-based propagation of prior knowledge to improve large-scale image analysis pipelines
Source: PLoS One. 2017 Nov 2;12(11):e0187535. doi: 10.1371/journal.pone.0187535 (PMC5667823; doi:10.1371/journal.pone.0187535)
Supplement: S1 Note — (PDF) [file pone.0187535.s001.pdf]

Fuzzy-based propagation of prior knowledge to improve large-scale image analysis pipelines

Johannes Stegmaier[1*], Ralf Mikut[1]

1 Institute for Applied Computer Science, Karlsruhe Institute of Technology, Eggenstein-Leopoldshafen, Germany

¤Current Address: Institute for Applied Computer Science, Karlsruhe Institute of Technology, Hermann-von-Helmholtz-Platz 1, 76344 Eggenstein-Leopoldshafen, Germany
* johannes.stegmaier@kit.edu

S1 Note: Fuzzy conjunction operators

In the methods section of the main paper, we introduced the concept of combining fuzzy set membership functions to a combined linguistic term using a general t-norm. To be a valid t-norm, an operator has to fulfill the following criteria [1]:

$$\cap(a,0) = 0 \quad \text{Zero Element} \tag{1}$$
$$\cap(a,1) = a \quad \text{Identity Element} \tag{2}$$
$$a \leq b \Rightarrow \cap(a,c) \leq \cap(b,c) \quad \text{Monotony} \tag{3}$$
$$\cap(a,b) = \cap(b,a) \quad \text{Commutativity} \tag{4}$$
$$\cap(a,\cap(b,c)) = \cap(\cap(a,b),c) \quad \text{Associativity} \tag{5}$$

There are three common choices for the conjunction operator (t-norm) that fulfill the above-mentioned criteria: the minimum operator: $\cap_{\min}(a,b) = \min(a,b)$, the bounded difference: $\cap_{\text{bd}}(a,b) = \max(0, a+b-1)$ and the algebraic product operator: $\cap_{\text{prod}}(a,b) = a \cdot b$. The corresponding fuzzy disjunction operators (t-conorms) are the maximum operator: $\cup_{\max}(a,b) = \max(a,b)$, the bounded sum: $\cup_{\text{bs}}(a,b) = \min(a+b,1)$ and the algebraic sum operator: $\cup_{\text{as}}(a,b) = a+b-a\cdot b$, respectively.

No perfect operator pair exists and not all rules known from binary logic are fulfilled by all operator pairs. For instance, idempotency ($\cap(a,a) = a$) is only fulfilled by the minimum operator and the law of non-contradiction ($\cap(a,1-a) = 0$) is only fulfilled by the bounded difference [1]. Furthermore, the product operator tends to underestimate the FSMD values for correlated features, where the minimum operator simply yields the lowest observed FSMD value. A non-zero value obtained by the minimum operator directly represents the lowest FSMD value of the considered fuzzy sets and can be used to identify the most likely error source. Therefore, instead of the multiplication-based conjunction we used in [2], we decided to use the minimum-based formulation in order to increase the interpretability of the combined feature membership value. However, it should be noted that we only consider one positive class and its complement (*i.e.*, *valid cell nucleus* and *not a valid cell nucleus*) throughout the present paper. Thus, inconsistencies caused by incomplete rule sets are avoided. If multiple positive classes are used, it is crucial to have a complete set of non-overlapping rules (all allowed combinations of input values should be represented in at least one rule), all fuzzy sets should be defined using a standard partition and the operator pair product/bounded sum should be used as a t-norm/t-conorm. For further details on general fuzzy set theory we refer the user to [3].

References

1. Gupta MM, Qi J. Theory of T-norms and Fuzzy Inference Methods. Fuzzy Sets and Systems. 1991;40(3):431–450.

2. Stegmaier J, Khan A, Reischl M, Mikut R. Challenges of Uncertainty Propagation in Image Analysis. In: Proc., 22. Workshop Computational Intelligence, Dortmund; 2012. p. 55–69.

3. Zimmermann HJ. Fuzzy Set Theory. Wiley Interdisciplinary Reviews: Computational Statistics 23. 2010; p. 317–332.
