# Supplementary material for: Fuzzy-based propagation of prior knowledge to improve large-scale image analysis pipelines
Source: PLoS One. 2017 Nov 2;12(11):e0187535. doi: 10.1371/journal.pone.0187535 (PMC5667823; doi:10.1371/journal.pone.0187535)
Supplement: S2 Note — (PDF) [file pone.0187535.s002.pdf]

Fuzzy-based propagation of prior knowledge to improve large-scale image analysis pipelines

Johannes Stegmaier[1*], Ralf Mikut[1]

1 Institute for Applied Computer Science, Karlsruhe Institute of Technology, Eggenstein-Leopoldshafen, Germany

¤Current Address: Institute for Applied Computer Science, Karlsruhe Institute of Technology, Hermann-von-Helmholtz-Platz 1, 76344 Eggenstein-Leopoldshafen, Germany
* johannes.stegmaier@kit.edu

S2 Note: Validation benchmark

To provide a thorough validation of the entire pipeline comprised of seed point detection, segmentation, multiview fusion and tracking, we used our recently presented approach for generating comprehensive validation benchmarks [1] with an adapted object movement simulation. In brief, object locations, object movements and object interactions were simulated over multiple time points to obtain movement behaviors that resembled biological specimens. At each simulated position, a video snippet containing a simulated fluorescent cellular nucleus [2] was added to an artificial 3D image, to form the data basis for each time point. To simulate acquisition deficiencies, the simulated images were disrupted by additive Gaussian noise, Poisson shot noise, a point-spread-function simulation [3], light attenuation and multiview acquisition simulation as detailed in [1].

For the sake of simplicity, we simulated objects that were moving on a spherical surface. Instead of using object displacements of a real embryo as described in [1], the simulated objects only moved due to density changes and the resulting repulsive and adhesive forces acting between neighboring objects. Furthermore, the simulation was constrained to a spherical surface to prevent arbitrary movement in the simulation space. A schematic illustration of the simulated specimen and an overview of the involved simulation steps of the benchmark is shown in S1 Fig. The repulsive ($\Delta\mathbf{x}^{\text{rep}}$) and adhesive forces ($\Delta\mathbf{x}^{\text{adh}}$) as well as the parameterization was taken from [5] and the boundary constraint was defined as:

$$\Delta\mathbf{x}^{\text{bdr}}(\mathbf{x},\mathbf{c},r_i,r_o,a) = \begin{cases} \frac{\mathbf{x}-\mathbf{c}}{\|\mathbf{x}-\mathbf{c}\|}\cdot\left(1-\frac{1}{e^{-a(\|\mathbf{x}-\mathbf{c}\|-r_i)}}\right), & \|\mathbf{x}-\mathbf{c}\| < r_i \\ -\frac{\mathbf{x}-\mathbf{c}}{\|\mathbf{x}-\mathbf{c}\|}\cdot\left(1-\frac{1}{e^{a(\|\mathbf{x}-\mathbf{c}\|-r_o)}}\right), & \|\mathbf{x}-\mathbf{c}\| > r_o \\ \mathbf{0}, & \text{else.} \end{cases} \quad (1)$$

In Eq (1), $\mathbf{x}$ is the centroid of the considered object, $\mathbf{c}$ is the center of the bounding volumes, r_i and r_o are the radii of the inner and the outer sphere, respectively, and finally a controls the shape of the sigmoidal boundary potential function. $\Delta\mathbf{x}^{\text{bdr}}$ only contributed to the displacement of a simulated object if the object was already out of the boundary. This additional displacement component prevented objects from entering the inner bounding sphere and from escaping the outer bounding sphere of the simulated embryo (S1 Fig A). Analogous to the formulation used in [1], the total displacement vector of a single object at a given time point can be summarized to:

$$\Delta \mathbf{x}_i^{\mathrm{tot}} = w_{\mathrm{bdr}} \cdot \Delta \mathbf{x}^{\mathrm{bdr}}(\mathbf{x}_i) + \sum_{j \in \{1,\ldots,N\}}^{i \neq j} \left[w_{\mathrm{rep}} \cdot \Delta \mathbf{x}^{\mathrm{rep}}(\|\mathbf{x}_i - \mathbf{x}_j\|) + w_{\mathrm{adh}} \cdot \Delta \mathbf{x}^{\mathrm{adh}}(\|\mathbf{x}_i - \mathbf{x}_j\|) \right]. \quad (2)$$

The weights of the adhesive and repulsive displacement components were set to the default values mentioned in [5]: $w_{\mathrm{adh}} = 0.52$, $w_{\mathrm{rep}} = 1.0$. Moreover, the weight for the boundary constraint $w_{\mathrm{bdr}} = 3.0$ was manually adjusted, such that the interacting objects remained within the spherical boundaries. Note that these parameters were empirically determined and that the presented model does not necessarily represent an accurate physical simulation of the interacting objects. However, the determined parameters produced movement behaviors that were similar to the epiboly movements observed during early zebrafish development due to increased object densities and boundary constraints that caused a directed movement on a sphere surface. Exemplary benchmark images are shown in S2 Fig for different time points and different additive Gaussian noise levels. Due to the availability of a complete ground truth, a detailed quantitative analysis of all involved pipeline steps could be performed with a single benchmark using the performance measures described in S3 Note. All acquisition deficiencies can be added to the simulated raw images using an XPIWIT pipeline [4]. The acquisition simulation pipeline, ground truth label images as well as the simulated raw images can be `https://bitbucket.org/jstegmaier/embryomicsbenchmark/downloads/PLOSONEValidationBenchmarks.zip`.

References

1. Stegmaier J, Arz J, Schott B, Otte JC, Kobitski A, Nienhaus GU, et al. Generating Semi-Synthetic Validation Benchmarks for Embryomics. In: Proc., IEEE International Symposium on Biomedical Imaging: From Nano to Macro; 2016.

2. Svoboda D, Ulman V. Generation of Synthetic Image Datasets for Time-lapse Fluorescence Microscopy. In: Image Analysis and Recognition. Berlin Heidelberg: Springer; 2012. p. 473–482.

3. Preibisch S, Amat F, Stamataki E, Sarov M, Singer RH, Myers E, et al. Efficient Bayesian-based Multiview Deconvolution. Nature Methods. 2014;11(6):645–648.

4. Bartschat A, Hübner E, Reischl M, Mikut R, Stegmaier J. XPIWIT - An XML Pipeline Wrapper for the Insight Toolkit. Bioinformatics. 2016;32(2):315–317.

5. Macklin P, Edgerton ME, Thompson AM, Cristini V. Patient-Calibrated Agent-Based Modelling of Ductal Carcinoma In Situ (DCIS): From Microscopic Measurements to Macroscopic Predictions of Clinical Progression. Journal of Theoretical Biology. 2012;301:122–140.
