# Supplementary material for: Fuzzy-based propagation of prior knowledge to improve large-scale image analysis pipelines
Source: PLoS One. 2017 Nov 2;12(11):e0187535. doi: 10.1371/journal.pone.0187535 (PMC5667823; doi:10.1371/journal.pone.0187535)
Supplement: S3 Note — (PDF) [file pone.0187535.s003.pdf]

Fuzzy-based propagation of prior knowledge to improve large-scale image analysis pipelines

Johannes Stegmaier[1*], Ralf Mikut[1]

1 Institute for Applied Computer Science, Karlsruhe Institute of Technology, Eggenstein-Leopoldshafen, Germany

¤Current Address: Institute for Applied Computer Science, Karlsruhe Institute of Technology, Hermann-von-Helmholtz-Platz 1, 76344 Eggenstein-Leopoldshafen, Germany
* johannes.stegmaier@kit.edu

S3 Note: Performance assessment

Seed point detection

The seed detection quality was evaluated using the benchmark datasets `SBDE1`, `SBDE2` and `SBDE3` (S1 Table). The intersections of the detected seeds with the labeled ground truth image were calculated. True positives (TP) were counted as ground truth objects that contained at least one seed point. Seed points that were detected in background regions or redundant detections of ground truth objects were considered as false positives (FP). Ground truth objects that did not contain a seed point were counted as false negatives (FN). Using TP, FP and FN, recall, precision and the F-Score (harmonic mean of precision and recall) were calculated. For all true positives, the average distance to the centroids of the respective ground truth objects was additionally calculated.

Segmentation

The segmentation quality was assessed using the benchmark datasets `SBDE1`, `SBDE2` and `SBDE3` (S1 Table). As the provided ground truth of the benchmark contained the complete label images of each frame, a detailed quantitative assessment of the automatic segmentation quality could be performed. The set of segmentation validation measures proposed by Coelho *et al.* was used, namely the Rand index (RI), the Jaccard index (JI), the normalized sum of distances (NSD) and the Hausdorff metric (HM). A detailed description of the measures can be found in [1]. Topological errors produced by the automatic segmentation were separated into added, missing, split or merged objects. Besides the error counts, this topological information was used to define the number of false positives as the sum of split and added cells and analogously the false negatives as the sum of merged and missing cells. These values were then used to calculate recall, precision and F-Score.

Tracking

To assess the tracking quality, the `SBDE4` dataset was used (S1 Table). The comparison of the investigated algorithms was performed using the `TRA` measure as described by Maška *et al.* [2]. This measure was calculated by considering the tracking result as an acyclic oriented graph and by comparing this graph to the respective ground truth

graph. The inverted, weighted and normalized number of required changes to transform the automatically generated graph to the ground truth graph yielded the normalized TRA measure (higher values are better with 1 being ideal). As the centroids of all objects, the complete temporal association and the object ancestry was known for the simulated SBDE4 dataset, the required ground truth graph could directly be generated using this data. To obtain a more detailed view on the errors made by the respective tracking algorithms, the number of false positive detections, false negative detections, incorrect edges, missing edges, redundant edges and merged objects were counted. Detailed descriptions of the validation measures are provided in [2].

References

1. Coelho LP, Shariff A, Murphy RF. Nuclear Segmentation in Microscope Cell Images: A Hand-Segmented Dataset and Comparison of Algorithms. In: Proc., IEEE International Symposium on Biomedical Imaging: From Nano to Macro; 2009. p. 518–521.

2. Maška M, Ulman V, Svoboda D, Matula P, Matula P, Ederra C, et al. A Benchmark for Comparison of Cell Tracking Algorithms. Bioinformatics. 2014;30(11):1609–1617.
