# Supplementary material for: Fuzzy-based propagation of prior knowledge to improve large-scale image analysis pipelines
Source: PLoS One. 2017 Nov 2;12(11):e0187535. doi: 10.1371/journal.pone.0187535 (PMC5667823; doi:10.1371/journal.pone.0187535)
Supplement: S4 Note — (PDF) [file pone.0187535.s004.pdf]

Fuzzy-based propagation of prior knowledge to improve large-scale image analysis pipelines

Johannes Stegmaier[1*], Ralf Mikut[1]

1 Institute for Applied Computer Science, Karlsruhe Institute of Technology, Eggenstein-Leopoldshafen, Germany

¤Current Address: Institute for Applied Computer Science, Karlsruhe Institute of Technology, Hermann-von-Helmholtz-Platz 1, 76344 Eggenstein-Leopoldshafen, Germany
* johannes.stegmaier@kit.edu

S4 Note: Multiview fusion

A frequently used approach to partly overcome quality deficiencies in 3D microscopy that are caused by attenuation and scattering of light along the axial direction is the acquisition of multiple views from different perspectives [1, 2]. Such complementary image stacks of a specimen can be obtained either by using multiple oppositely arranged detection paths [2, 3] or by a rotation of the probe using a single camera [4]. Both strategies for the acquisition of multiview images require a subsequent fusion step of the information present in the individual views into a single consistent representation. The benchmark dataset `SBDE4` used for the tracking validation contains such a simulated multiview acquisition, *i.e.*, at each time point, two opposite images were simulated. To fuse to complementary views, we use a segmentation-based fusion approach that combines the results of separately applied segmentation on the different views to a single segmentation image. As the image transformation of 180° was already known from the benchmark generation step, the registration process was skipped. Corresponding segments were identified using a histogram-based approach, *i.e.*, a 2D label histogram was filled by iterating over all voxels of both 3D segmentation images and by successively increasing the 2D histogram bins indicated by the respective image label pairs. Segments that were only present in one or the other image could be found by searching for empty columns and rows of the histogram, respectively, and by copying these segments without any further consideration into the new result image. In the next step, the assignments of the remaining segments in both images were identified by searching for the respective label pair with the maximum overlap. In the case of segmentation fusion, it was not desired to perform a weighted average of the segments but rather to use the better segment for the new image. This was accomplished by simply selecting the segment with a higher FSMD value to the desired class of objects based on the FSMD values of the segments and the seed points that contained the information about the validity of the segment and the axial localization of the extracted objects, respectively. The final segmentation image was then used for tracking.

References

1. Krzic U, Gunther S, Saunders TE, Streichan SJ, Hufnagel L. Multiview Light-Sheet Microscope for Rapid *in toto* Imaging. Nature Methods. 2012;9(7):730–733.

2. Chhetri RK, Amat F, Wan Y, Höckendorf B, Lemon WC, Keller PJ. Whole-Animal Functional and Developmental Imaging with Isotropic Spatial Resolution. Nature Methods. 2015;12(12):1171—1178.

3. Tomer R, Khairy K, Amat F, Keller PJ. Quantitative High-Speed Imaging of Entire Developing Embryos with Simultaneous Multiview Light-Sheet Microscopy. Nature Methods. 2012; p. 755–763.

4. Kobitski A, Otte JC, Takamiya M, Schäfer B, Mertes J, Stegmaier J, et al. An Ensemble-Averaged, Cell Density-based Digital Model of Zebrafish Embryo Development Derived from Light-Sheet Microscopy Data with Single-Cell Resolution. Scientific Reports. 2015;5(8601):1–10.
