# Supplementary figures and images for: Fuzzy-based propagation of prior knowledge to improve large-scale image analysis pipelines
Source: PLoS One. 2017 Nov 2;12(11):e0187535. doi: 10.1371/journal.pone.0187535 (PMC5667823; doi:10.1371/journal.pone.0187535)

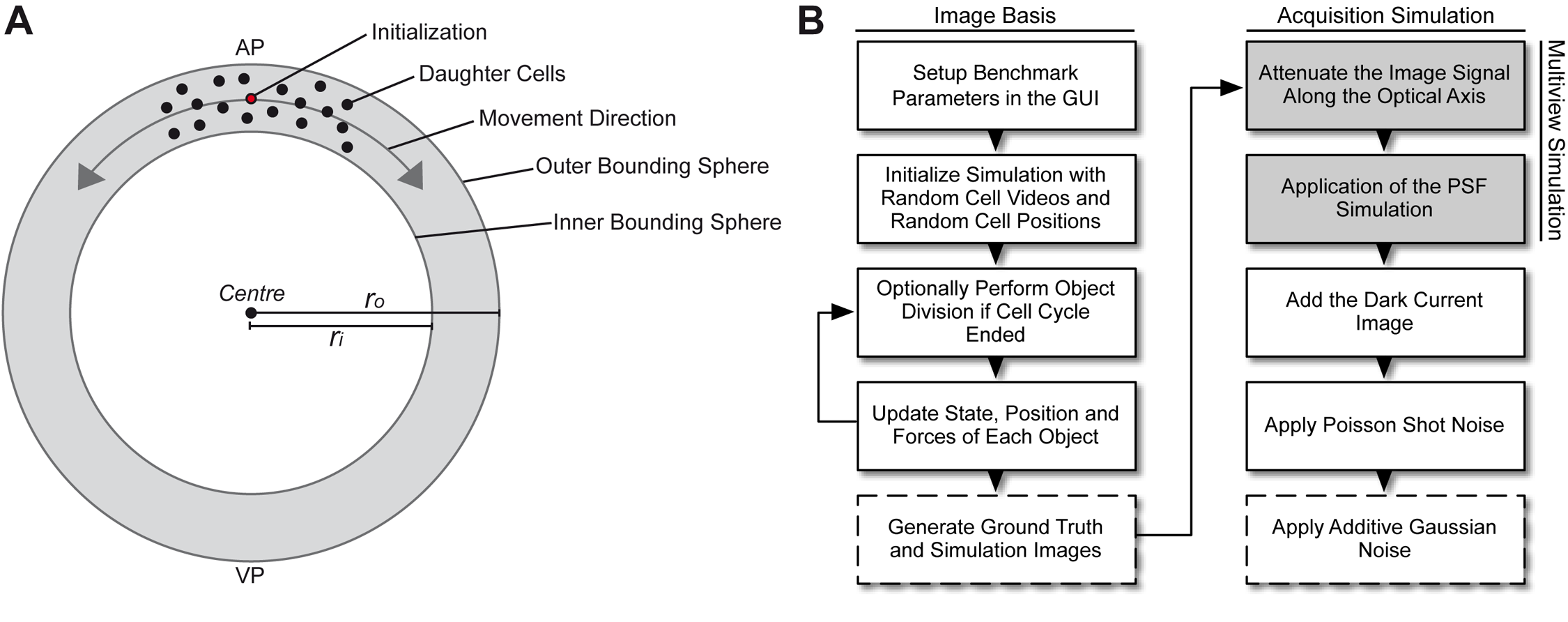

Supplement: S1 Fig — (A) Illustration of the embryo simulation. Starting with an initial object, multiple division cycles were simulated including object interaction, object divisions and morphological constraints. Due to inner and outer bounding spheres and the between-object interactions, cells migrated from the animal pole (AP) to the artificial vegetal pole (VP), which produced a behavior similar to the epiboly movement of zebrafish embryogenesis. (B) The performed steps for a realistic simulated 3D+t benchmark. The left column reflects the object simulation and returned raw images that contained dynamic objects and the associated ground truth data. The right column contains the acquisition simulation, which distorted the simulated images by an artificial signal attenuation, a point spread function simulation (PSF), a dark current image simulation, Poisson distributed photon shot noise and additive Gaussian noise. Steps shaded in gray could optionally consider image rotation, if a multiview experiment was simulated and the output operators are indicated by dashed edge lines (adapted from [38]). (TIF) [file pone.0187535.s005.tif]

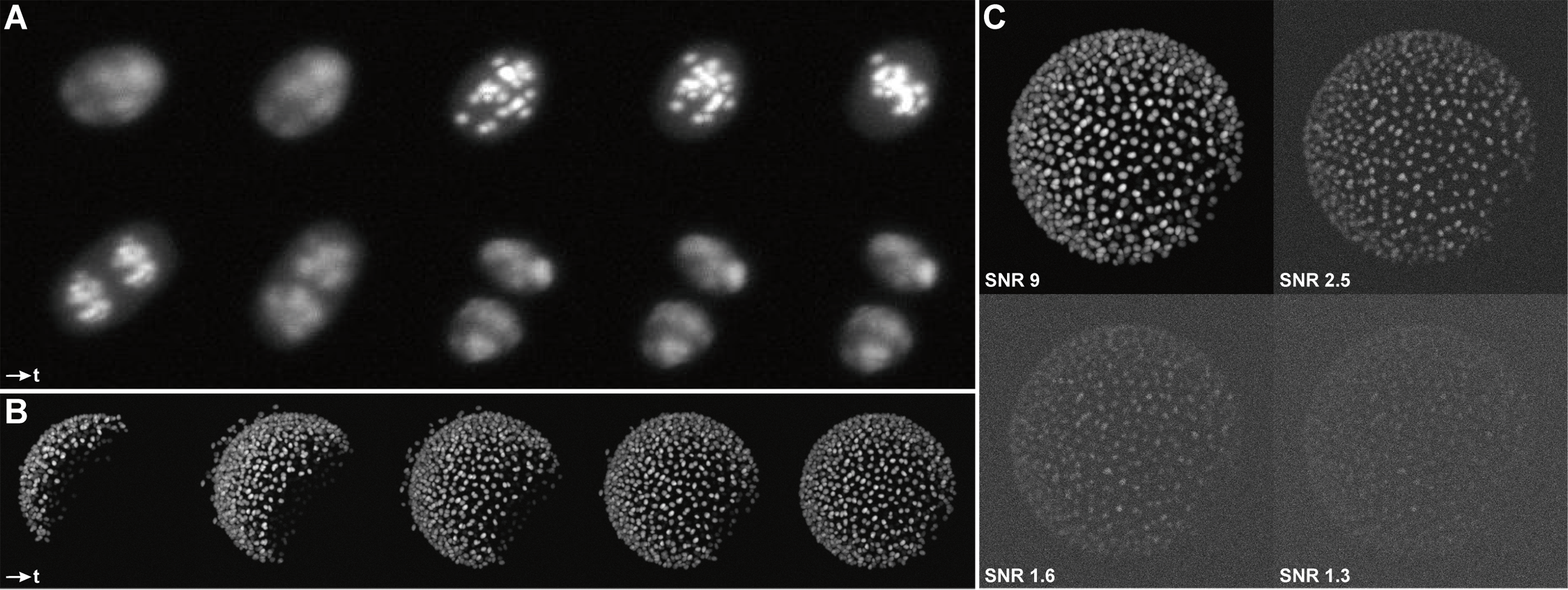

Supplement: S2 Fig — (A) Maximum projections of an extracted division cycle of one simulated nucleus [53]. Time increases from left to right and top to bottom. Single objects were randomly initialized and simulated for a predefined experimental duration. This approach yielded a simulated embryo including object movement, object interaction and object divisions with available ground truth (B). Generated raw image sequences were manipulated to simulate various acquisition conditions, such as different levels of additive Gaussian noise (C). The ground truth enabled a quantitative analysis of the algorithmic performance on realistic image data. (TIF) [file pone.0187535.s006.tif]

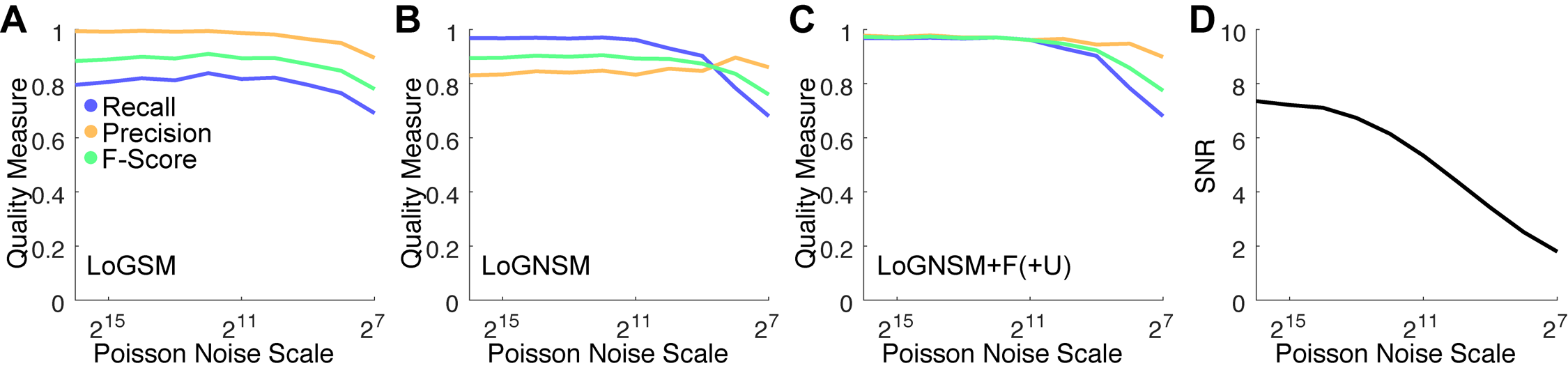

Supplement: S3 Fig — The performance measures recall, precision and F-Score are plotted versus the Poisson noise scale (inversely correlated with the noise level, see [54] for implementation details). The qualitative behavior matches the one observed for additive Gaussian noise. The influence of the Poisson noise scale on the signal-to-noise ratio of the images is visualized in (D). Optimal thresholds for the twmi parameter were identified using the interactive graphical user interface depicted in S6 Fig. Furthermore, examples for different parameter settings and the identified optimal parameters are shown in S7 Fig. (TIF) [file pone.0187535.s007.tif]

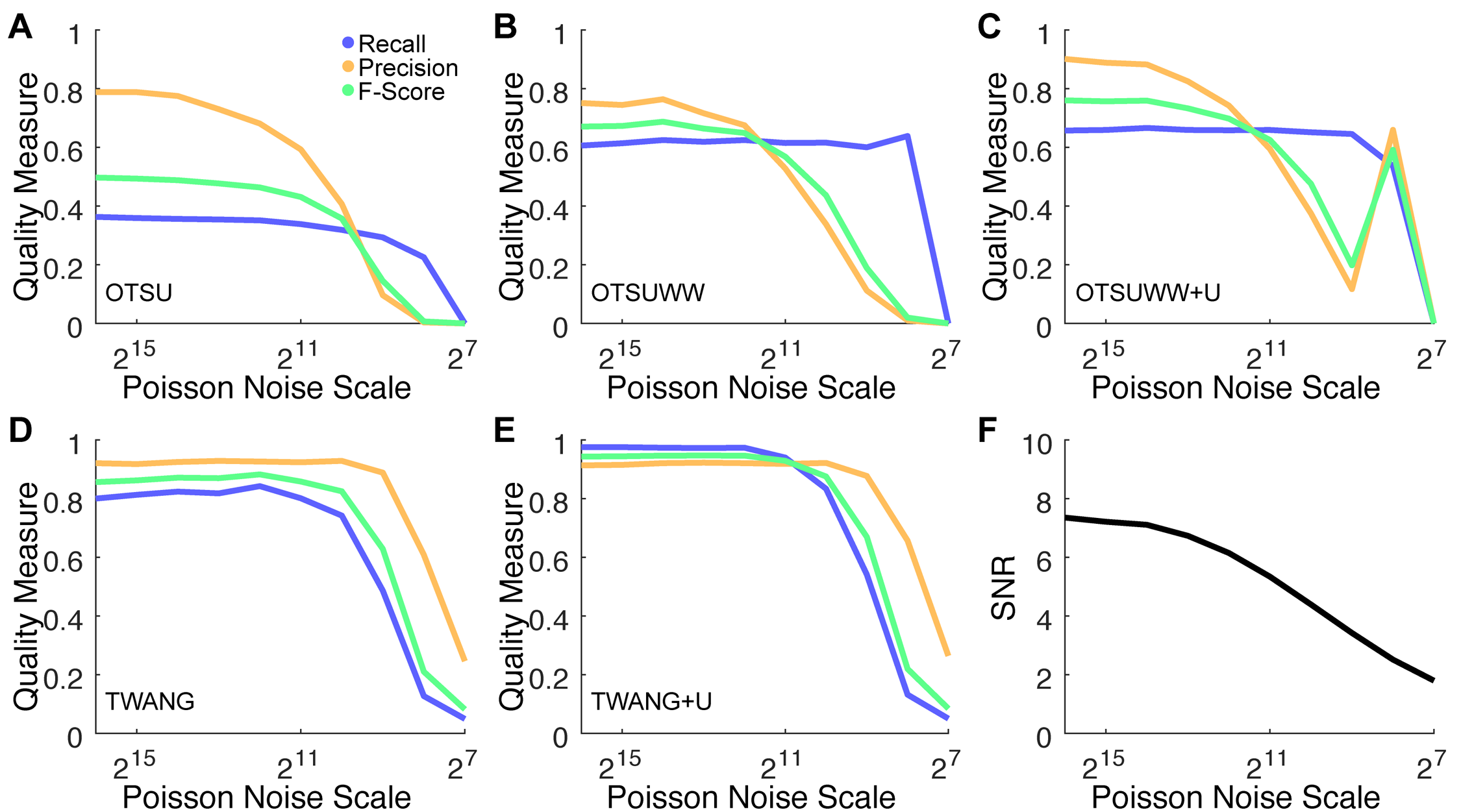

Supplement: S4 Fig — The methods based on adaptive thresholding suffered from high noise levels and produced a successively increased amount of false positive detections (OTSU, OTSUWW), which could be efficiently suppressed using the uncertainty framework-based extension (OTSUWW+U). The precision jump at the penultimate noise level observed for OTSUWW+U is actually not an artifact, but caused by a decreased amount of erroneous detections in the lower z-layers that fall below the minimum size criterion due to increased noise-related object splits (see column OTSUWW+U in Fig 7). However, it should be noted that such artificially high noise levels are unlikely to occur for real fluorescence microscopy images of stained cellular nuclei. Analogous to the results obtained for different additive Gaussian noise levels, the result quality of both TWANG versions directly correlated with the quality of the provided seed points, i.e., TWANG+U benefited from the improved detection rate of LoGNSM+F+U. (TIF) [file pone.0187535.s008.tif]

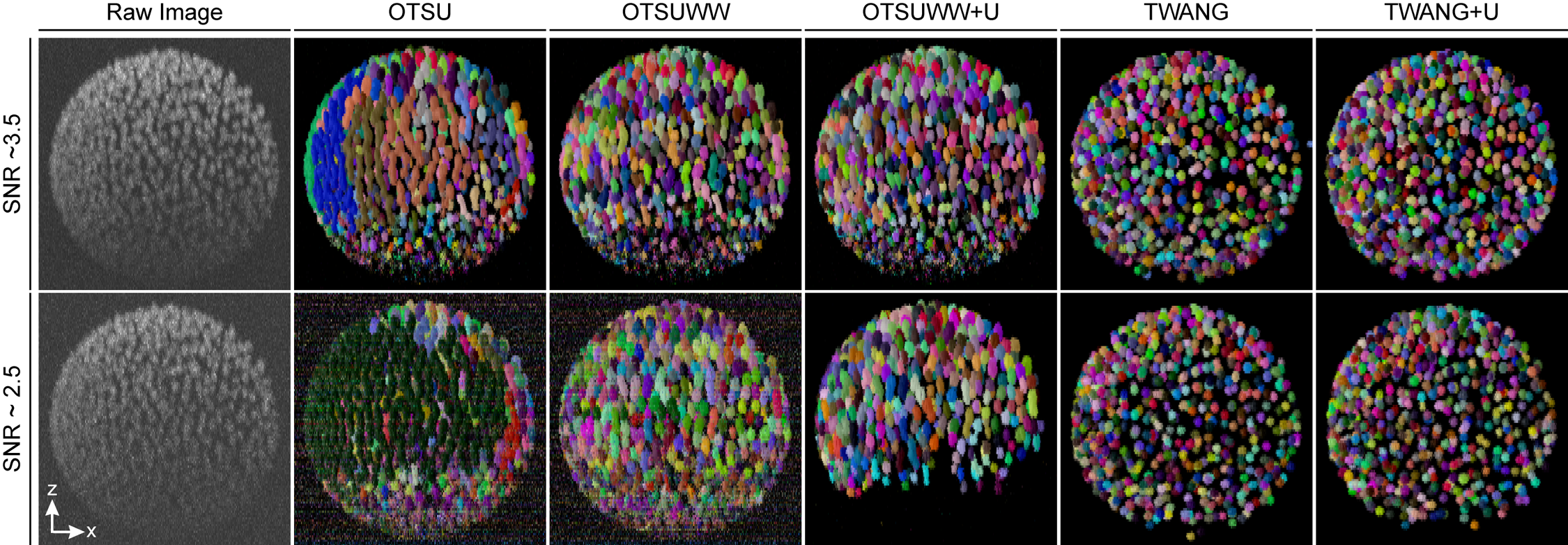

Supplement: S5 Fig — A random color-code was applied to visualize under- and over-segmentation errors. Both OTSU and OTSUWW did not produce satisfactory results with a lot of background noise detections and under-segmentation errors. The uncertainty-based improvements help to improve the quality for OTSUWW+U, and TWANG(+U) performed reasonably well depending on the quality of the provided seeds. (TIF) [file pone.0187535.s009.tif]

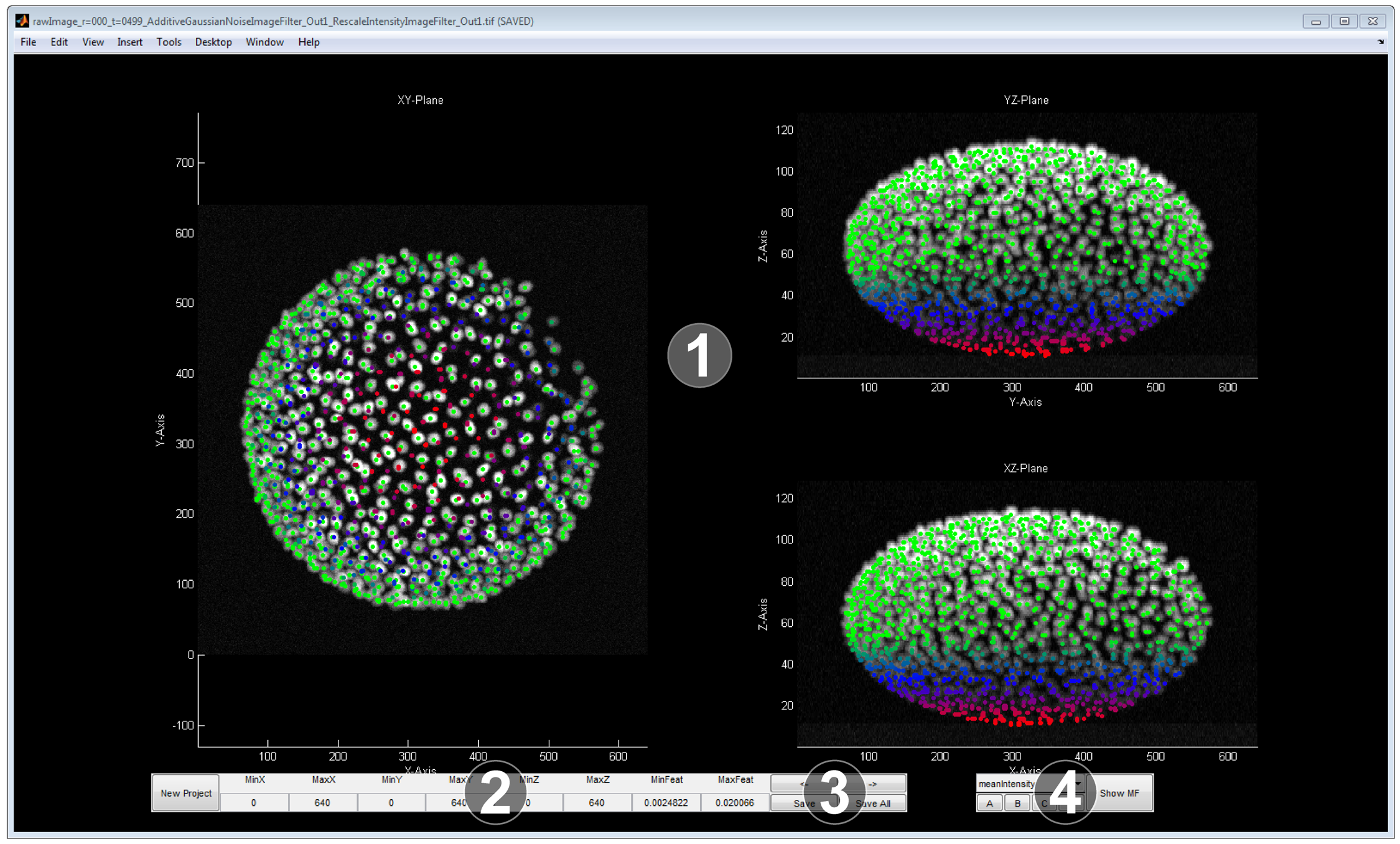

Supplement: S6 Fig — The three main panels depict the maximum intensity projection views of the XY, XZ and YZ plane of an image containing simulated cell nuclei (white objects) with superimposed centroids of detected objects (1). Undesired objects can be filtered based on the feature values, such as spatial constraints or intensity constraints (2). Settings can be propagated to all other images of the project to accelerate the workflow and filtered results can be saved as *.csv files for further processing (3). All parameters used in the uncertainty propagation framework, i.e., the fuzzy set membership parameterization of any of the available features can be performed in this GUI. The color-code from red (low FSMD) over blue (medium FSMD) to green (high FSMD) provides an instant visual feedback of the membership degree obtained for the individual objects and helps to identify the optimal settings (4). (TIF) [file pone.0187535.s010.tif]

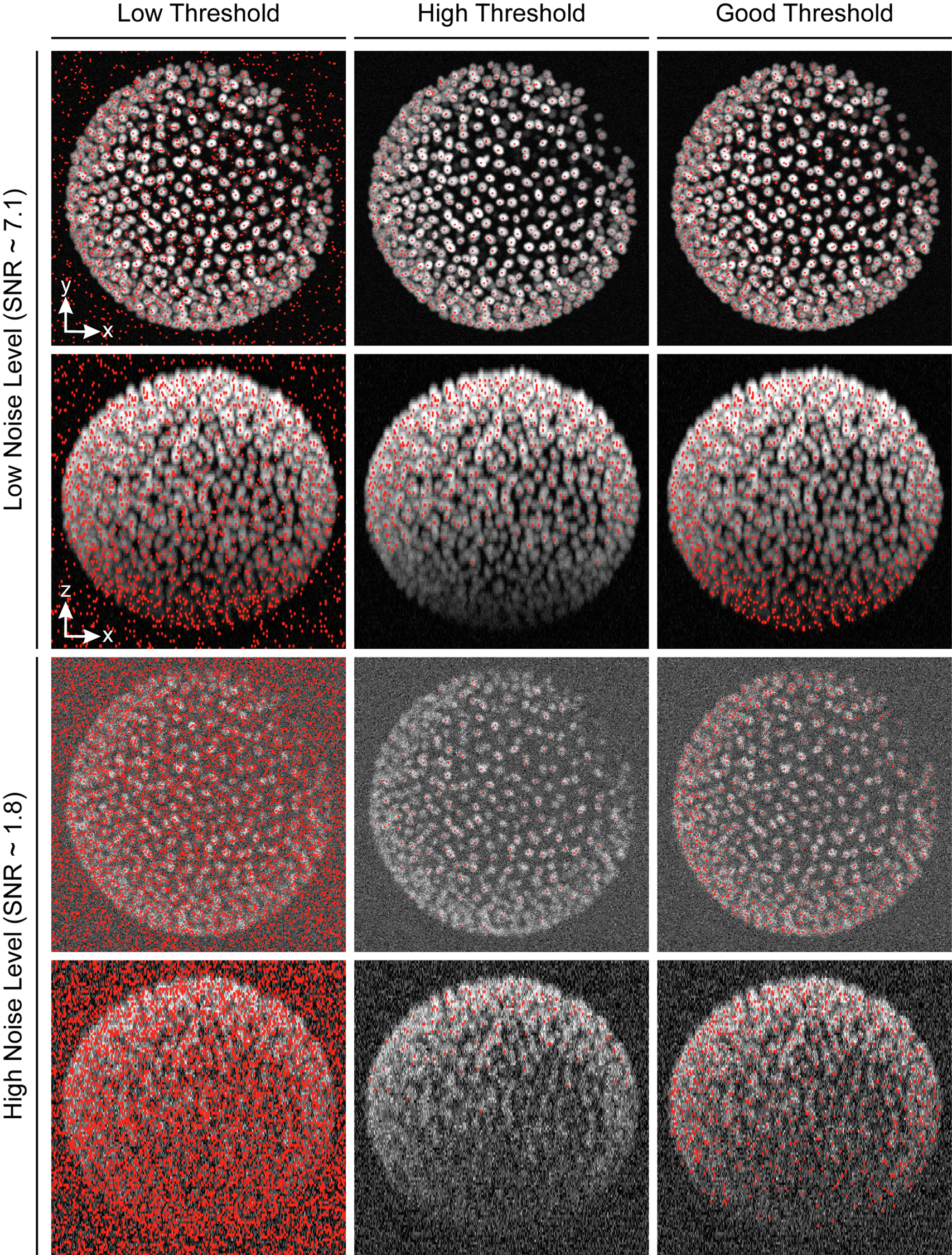

Supplement: S7 Fig — The columns show the filtered seed points for different settings of the twmi threshold parameter with a too low threshold (left column, many false positive detections), a too high threshold (center column, many false negatives) and a visually determined good threshold (right column, best visually observable balance of false positives and false negatives). For higher noise levels, finding an optimal threshold becomes ambiguous, due to low signal to noise ratio at lower z-slices. Thus, the selection of the threshold largely depends on the respective preference of the user and can cause small fluctuations in the quantitative evaluation for different noise levels (see Figs 6 and 7 in the main text as well as S3 and S5 Figs). (TIF) [file pone.0187535.s011.tif]

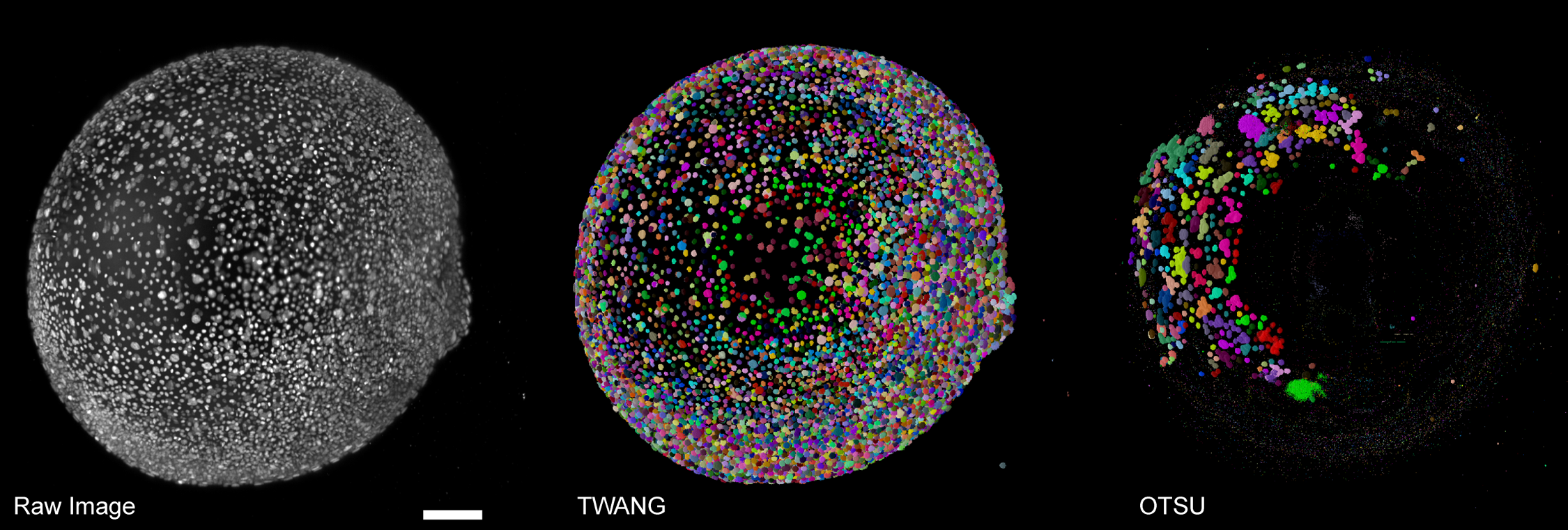

Supplement: S8 Fig — OTSU completely failed to extract meaningful information in this case and only captured a few correct nuclei by chance. TWANG could resolve most of the nuclei correctly and by design largely avoids under-segmentation errors. As the results provided by the plain OTSU algorithm were not properly extracting the embryonic shape, the watershed-based post-processing steps used in OTSUWW and OTSUWW+U were skipped. The results of TWANG+U are visually indiscernible from the plain TWANG results and we thus only show one panel for convenience. Furthermore, due to the image size of about 5 GB per frame, applying the 3D watershed algorithm on the entire image was not possible given the memory limitations of 32 GB on our test computer. A lack of ground truth for these large-scale data sets only allowed a qualitative comparison (see [39] for details). Scale bar: 100μm. (TIF) [file pone.0187535.s012.tif]
