# Supplementary material for: Fuzzy-based propagation of prior knowledge to improve large-scale image analysis pipelines
Source: PLoS One. 2017 Nov 2;12(11):e0187535. doi: 10.1371/journal.pone.0187535 (PMC5667823; doi:10.1371/journal.pone.0187535)
Supplement: S1 Table — (PDF) [file pone.0187535.s013.pdf]

Fuzzy-based propagation of prior knowledge to improve large-scale image analysis pipelines

Johannes Stegmaier[1*], Ralf Mikut[1]

1 Institute for Applied Computer Science, Karlsruhe Institute of Technology, Eggenstein-Leopoldshafen, Germany

¤Current Address: Institute for Applied Computer Science, Karlsruhe Institute of Technology, Hermann-von-Helmholtz-Platz 1, 76344 Eggenstein-Leopoldshafen, Germany

* johannes.stegmaier@kit.edu

S1 Table: Nomenclature, abbreviations and symbols.

In this supplementary note, the notation used throughout the manuscript is summarized. The initial enumeration demonstrates the basic concepts used for nomenclature and the subsequent list of symbols explains all used symbols within their context. The nomenclature is based on the formulation given in [1] and the list of symbols represents a shortened version of the list provided in [2].

- Lower case letters are used for scalars, parameters, indexing variables and functions (*e.g.*, a, b, c, d are common names for parameters, $f(\cdot), g(\cdot), h(\cdot)$ are common names for functions or functionals, i, j, k, l are common names for indices and finally x, y, z are common names for scalars or variables)
- Upper case letters are used for number of entities (*e.g.*, N, N_f for the number of data tuples and the number of features, respectively)
- Upper case bold face letters represent matrices (*e.g.*, $\mathbf{M}, \mathbf{N}$)
- Lower case bold face letters represent vectors (*e.g.*, $\mathbf{x}, \mathbf{y}$ for column vectors and $\mathbf{x}^\top, \mathbf{y}^\top$ for row vectors)
- Upper case script and Greek letters represent sets (*e.g.*, $\Omega, \Theta, \mathcal{X}, \mathcal{L}$)
- Accessing elements of data structures is denoted by square brackets (*e.g.*, $\mathbf{x}[1]$ accesses the first element of vector $\mathbf{x}$, $\mathbf{M}[1, 2]$ accesses the matrix element in the first row and second column)
- Elements topped with a hat refer to estimates of a certain quantity (*e.g.*, $\hat{x}$ represents an estimate of the scalar x, $\hat{\mathbf{x}}$ represents an estimate of vector $\mathbf{x}$)
- Elements topped with a tilde refer to a changed quantity (*e.g.*, $\tilde{\mathcal{X}}$ represents a modified subset of $\mathcal{X}$)
- Optimal solutions are indicated by an asterisk (*e.g.*, x^* would denote the optimal solution to $\min(f(x))$)

Symbol	Description
2D	Two dimensional
2D+t	Two spatial dimensions and temporal dimension, e.g. 2D videos
3D	Three dimensional
3D+t	Three spatial dimensions and temporal dimension, e.g. 3D videos
$\mathbf{c}$	Centroid of an object
FSMD	**F**uzzy **S**et **M**embership **D**egree
HM	**H**ausdorff **M**etric
hpf	**H**ours **P**ost **F**ertilization (used to specify the age of zebrafish embryos)
i, j, k, l, m, n	Index variables defined by the context
$\mathbf{I}$	General image variable
JI	**J**accard **I**ndex
l	Index variable for linguistic terms
LoG	**L**aplacian-**o**f-**G**aussian
LoGSM	Seed detection in the LoG scale space maximum projection using a strict maximum detection
LoGNSM	Seed detection in the LoG scale space maximum projection using a non-strict maximum detection
LoGNSM+F	Same as LoGNSM but with additional fusion (F) of redundant detections
LoGNSM+F+U	Same as LoGNSM but with uncertainty-based threshold (U) and the fusion of LoGNSM+F
MBF	Abbreviation for **m**em**b**ership **f**unction
n	Index variable for data tuples
N	General number of data tuples, objects or dimensions specified by the context
N_{cl}	General number of combined linguistic terms
$N_{\mathrm{cl},i}$	Number of combined linguistic terms for operator i
N_{f}	General number of features
$N_{\mathrm{f},i}$	Number of extracted features produced by operator i (i.e. columns in the result matrix)
N_i	Number of data tuples produced by operator i (i.e. rows in the result matrix)
N_{l}	General number of linguistic terms
$N_{\mathrm{l},i}$	Number of linguistic terms used by operator i
N_{op}	Number of operators within an analysis pipeline
N_{p}	Dimensionality of the a parameter vector, *i.e.*, the number of parameters
$\mathcal{N}(\mu, \sigma)$	Normally distributed random variable with mean μ and standard deviation σ
*+NN	Nearest neighbor-based tracking applied on the algorithm specified by *
NSD	**N**ormalized **S**um of **D**istances
OTSU	Adaptive thresholding using Otsu's method [3]
OTSUWW	Same segmentation as OTSU including a watershed-based object splitting
OTSUWW+U	Same segmentation as OTSU using uncertainty-based object splitting and noise removal

PSF	**P**oint **S**pread **F**unction
$P_\lambda(.)$	Poisson process to simulate photon shot noise using the image intensities as mean
$q_\mathrm{l}, q_\mathrm{u}$	Parameters for lower and upper quantile saturation
r	General variable for radius
r_i, r_o	Radius of the inner and outer sphere that are used for the validation benchmark
RI	**R**and **I**ndex
SBDE	**S**imulated **B**enchmark **D**ataset **E**mbryo
SNR	**S**ignal-to-**N**oise **R**atio
t_dbc	Distance-based cutoff value used for feature fusion
t_wmi	Threshold for the window mean intensity feature
TWANG	**T**hreshold of **W**eighted Intensity **A**nd Seed-**N**ormal **G**radient Dot Product Image segmentation as described in [4]
TWANG+U	Same as TWANG segmentation but using LoGNSM+F+U for seed detection
$w_\mathrm{adh}, w_\mathrm{rep}, w_\mathrm{bdr}$	Weights for the adhesive displacement, the repulsive displacement and the boundary displacement
x	General variable defined by the context
$\mathbf{x}$	General vector variable defined by the context
$x_i[n, l]$	l-th feature in the n-th data tuple produced by the i-th operator
$\mathbf{x}_i[n]$	n-th data tuple produced by the i-th operator containing $N_{f,i}$ feature entries
$\mathbf{X}_i$	Result matrix of the i-th operator containing N_i data tuples with $N_{f,i}$ features
$\mathcal{X}_i$	Variable for an output set of operator i
XPIWIT	**X**ML **Pi**peline **W**rapper for the **I**nsight **T**oolkit
α_{il}	Forward threshold of operator i and linguistic term l
β_{il}	Backwards threshold of operator i and linguistic term l
$\Delta\mathbf{x}^\mathrm{adh}$	Adhesive displacement vector of two neighboring objects
$\Delta\mathbf{x}^\mathrm{bdr}$	Boundary constraint for dynamic object simulation
$\Delta\mathbf{x}^\mathrm{rep}$	Repulsive displacement vector of two neighboring objects
$\Delta\mathbf{x}^\mathrm{tot}$	Sum of all displacement vectors of a single object
θ	General parameter specified in the context
$\boldsymbol{\theta}$	General parameter vector specified in the context
μ	Arithmetic mean value
$\mu_\mathcal{A}(\cdot)$	Membership function for the fuzzy set $\mathcal{A}$
$\boldsymbol{\mu}_{\mathcal{A}_l}(\cdot)$	Vector of membership functions for all features of the l-th linguistic term
$\boldsymbol{\mu}_{\mathcal{A}_{il}}(\cdot)$	Vector of membership functions for all features of the l-th linguistic term of processing operator i
$\mu_{A_{ifl}}(\cdot)$	Membership function of feature f and linguistic term l of the i-th processing operator
μ_fg	Mean intensity of the foreground signal (used for SNR calculation)
σ	Standard deviation
σ^2	Variance
σ_agn	Zero-mean additive Gaussian noise standard deviation
σ_bg	Standard deviation of the background signal of an image (used for SNR calculation)

σ_{grad}	TWANG segmentation regularization scale used to smooth the vector field
σ_{kernel}	TWANG segmentation weighting kernel standard deviation
σ_{min}	Minimum scale used for the LoG-based seed detection
σ_{max}	Maximum scale used for the LoG-based seed detection
σ_{smooth}	The standard deviation of a Gaussian smoothing kernel
σ^2_{smooth}	The variance of a Gaussian smoothing kernel
σ_{step}	Step size between adjacent scales used for the LoG-based seed detection
Φ	Variable for a set
$\chi_{\mathcal{C}}(x)$	Characteristic function of the set $\mathcal{C}$
ω_{kpm}	TWANG segmentation weighting kernel plateau multiplier
Ω_i	Variable for set that combines subsets of the outputs of operator i and operator $i-1$
$\tilde{\Omega}_i$	Variable for a modified input set of operator $i+2$

References

1. Mikut R. Data Mining in der Medizin und Medizintechnik. Habilitationsschrift, Universitätsverlag Karlsruhe; 2008.

2. Stegmaier J. New Methods to Improve Large-Scale Microscopy Image Analysis with Prior Knowledge and Uncertainty. Karlsruhe Institute of Technology; 2016.

3. Otsu N. A Threshold Selection Method from Gray-Level Histograms. IEEE Transactions on Systems, Man and Cybernetics. 1979;9:62–66.

4. Stegmaier J, Otte JC, Kobitski A, Bartschat A, Garcia A, Nienhaus GU, et al. Fast Segmentation of Stained Nuclei in Terabyte-Scale, Time Resolved 3D Microscopy Image Stacks. PLoS ONE. 2014;9(2):e90036.
