# Supplementary material for: Fuzzy-based propagation of prior knowledge to improve large-scale image analysis pipelines
Source: PLoS One. 2017 Nov 2;12(11):e0187535. doi: 10.1371/journal.pone.0187535 (PMC5667823; doi:10.1371/journal.pone.0187535)
Supplement: S2 Table — Columns list the dataset identifier (Name), the number of images (Im.), the number of noise levels (N.), the number of objects (Objects), the image resolution, the noise parameter ranges for σagn and the dataset description. (PDF) [file pone.0187535.s014.pdf]

Fuzzy-based propagation of prior knowledge to improve large-scale image analysis pipelines

Johannes Stegmaier[1*], Ralf Mikut[1]

1 Institute for Applied Computer Science, Karlsruhe Institute of Technology, Eggenstein-Leopoldshafen, Germany

¤Current Address: Institute for Applied Computer Science, Karlsruhe Institute of Technology, Hermann-von-Helmholtz-Platz 1, 76344 Eggenstein-Leopoldshafen, Germany
* johannes.stegmaier@kit.edu

S2 Table: The benchmark datasets used for the validation experiments.

Name	Im.	N.	Objects	Resolution	Noise Level	Description
`SBDE1`	10	1	300–1000	640×640×128	$\sigma_{\mathrm{agn}} = 0.0007$	Five pairs of subsequent frames with different numbers of objects. Used for seed detection and segmentation validation.
`SBDE2`	20	20	1000	640×640×128	$\sigma_{\mathrm{agn}} \in [0.0005, 0.01]$	Single frame of `SBDE1` with 20 different levels of Gaussian noise. Used for seed detection and segmentation validation.
`SBDE3`	10	10	1000	640×640×128	scale $\in [2^7, 2^{16}]$	Single frame of `SBDE1` with 10 different levels of Poisson noise. Used for seed detection and segmentation validation. See [1] for implementation details of the Poisson noise.
`SBDE4`	100	1	1000	640×640×128	$\sigma_{\mathrm{agn}} = 0.0007$	Sequential time points containing 1000 objects each. Images are rotated by 180° at every frame to simulate a simultaneous acquisition multiview experiment SiMV (2×50 images). Used for the validation of the tracking.

Columns list the dataset identifier (Name), the number of images (Im.), the number of noise levels (N.), the number of objects (Objects), the image resolution, the noise parameter ranges for σ_{agn} and the dataset description.

References

1. Lehmann G. Noise Simulation. The Insight Journal. 2010; p. 1–7.
