# Supplementary material for: Fuzzy-based propagation of prior knowledge to improve large-scale image analysis pipelines
Source: PLoS One. 2017 Nov 2;12(11):e0187535. doi: 10.1371/journal.pone.0187535 (PMC5667823; doi:10.1371/journal.pone.0187535)
Supplement: S3 Table — (PDF) [file pone.0187535.s015.pdf]

Fuzzy-based propagation of prior knowledge to improve large-scale image analysis pipelines

Johannes Stegmaier[1*], Ralf Mikut[1]

1 Institute for Applied Computer Science, Karlsruhe Institute of Technology, Eggenstein-Leopoldshafen, Germany

¤Current Address: Institute for Applied Computer Science, Karlsruhe Institute of Technology, Hermann-von-Helmholtz-Platz 1, 76344 Eggenstein-Leopoldshafen, Germany
* johannes.stegmaier@kit.edu

S3 Table: Abbreviations, parameterizations and descriptions of the investigated seed detection algorithms.

Method	Parameters	Description
LoGSM	$\sigma_{\text{min}} = 4$, $\sigma_{\text{max}} = 8$, $\sigma_{\text{step}} = 1$, $t_{\text{wmi}} = 0.0025$	Seed detection in the LoG scale space maximum projection with a manually adjusted window mean intensity threshold (t_{wmi}) and a strict maximum detection.
LoGNSM	$\sigma_{\text{min}} = 4$, $\sigma_{\text{max}} = 8$, $\sigma_{\text{step}} = 1$, $t_{\text{wmi}} = 0.0025$	Seed detection in the LoG scale space maximum projection with a manually adjusted window mean intensity threshold (t_{wmi}) and a non-strict maximum detection.
LoGNSM+F	$\sigma_{\text{min}} = 4$, $\sigma_{\text{max}} = 8$, $\sigma_{\text{step}} = 1$, $t_{\text{wmi}} = 0.0025$, $t_{\text{dbc}} = 5$	Same detection as LoGNSM but with additional fusion (F) of redundant detections using a hierarchical clustering approach with a distance-based cutoff value (t_{dbc}).
LoGNSM+F+U	$\sigma_{\text{min}} = 4$, $\sigma_{\text{max}} = 8$, $\sigma_{\text{step}} = 1$, $t_{\text{dbc}} = 5$, $\boldsymbol{\theta}_{\text{wmi}} = (0.0025, 0.0025, \infty, \infty)$, $\boldsymbol{\theta}_{\text{smi}} = (0.0007, 0.0007, \infty, \infty)$, $\boldsymbol{\theta}_{\text{zpos}} = (50, 250, \infty, \infty)$, $\alpha_{11} = 0.0001$, $\beta_{11} = \alpha_{11}$	Same detection as LoGNSM but with uncertainty-based (U) threshold and the fusion of LoGNSM+F. The forward threshold α_{11} is set slightly above zero, such that obvious false positives are rejected. As no further processing was needed β_{11} was set to α_{11}.
