# Supplementary material for: Fuzzy-based propagation of prior knowledge to improve large-scale image analysis pipelines
Source: PLoS One. 2017 Nov 2;12(11):e0187535. doi: 10.1371/journal.pone.0187535 (PMC5667823; doi:10.1371/journal.pone.0187535)
Supplement: S4 Table — (PDF) [file pone.0187535.s016.pdf]

Fuzzy-based propagation of prior knowledge to improve large-scale image analysis pipelines

Johannes Stegmaier[1*], Ralf Mikut[1]

1 Institute for Applied Computer Science, Karlsruhe Institute of Technology, Eggenstein-Leopoldshafen, Germany

¤Current Address: Institute for Applied Computer Science, Karlsruhe Institute of Technology, Hermann-von-Helmholtz-Platz 1, 76344 Eggenstein-Leopoldshafen, Germany
* johannes.stegmaier@kit.edu

S4 Table: Abbreviations, parameters and descriptions of the segmentation algorithms.

Method	Parameters	Description
OTSU	$\sigma_{\text{smooth}} = 1$	Adaptive thresholding using Otsu's method applied on a regularized raw input image [1].
OTSUWW	$\sigma_{\text{smooth}} = 1$	Same segmentation as OTSU. Merged regions in the entire image were split based on cleaned seeds of the LoGSM method, using a seeded watershed approach.
OTSUWW+U	$\sigma_{\text{smooth}} = 1$, $\alpha_{21} = 0.1$, $\beta_{21} = 0.1$	Same segmentation as OTSU. Small noise objects with FSMD values below $\alpha_{21} = 0.1$ were rejected from further processing. Merged regions with FSMD below $\beta_{21} = 0.1$ that were larger than expected were split locally and in parallel using the cleaned and fused seeds of the LoGNSM+F+U method and a seeded watershed approach in small cropped regions.
TWANG	$\sigma_{\text{grad}} = 3$, $\sigma_{\text{kernel}} = 3$, $\omega_{\text{kpm}} = 1.41$	TWANG segmentation as described in [2] using the cleaned seeds provided by the LoGSM method. The manually optimized parameter $\omega_{\text{kpm}} = 1.41$ yielded better results than the default value of 1.0 for this dataset.
TWANG+U	$\sigma_{\text{grad}} = 3$, $\sigma_{\text{kernel}} = 3$, $\omega_{\text{kpm}} = 1.41$, $\alpha_{21} = 0$, $\beta_{21} = 0$	TWANG segmentation as described in [2] using the cleaned and fused seeds provided by the LoGNSM+F+U method. $\alpha_{21} = 0$, $\beta_{21} = 0$ are adjusted such that all segments are propagated unchanged.

References

1. Otsu N. A Threshold Selection Method from Gray-Level Histograms. IEEE Transactions on Systems, Man and Cybernetics. 1979;9:62–66.

2. Stegmaier J, Otte JC, Kobitski A, Bartschat A, Garcia A, Nienhaus GU, et al. Fast Segmentation of Stained Nuclei in Terabyte-Scale, Time Resolved 3D Microscopy Image Stacks. PLoS ONE. 2014;9(2):e90036.
